# Thickness‐Gradient Liquid Metal Connectors for Seamless Electrophysiological Sensors

**DOI:** 10.1002/advs.77844

**Published:** 2026-10-16

**Authors:** Jinyoung Kim, Jaejun Lee, Christopher E. Tabor, Vladimir V. Tsukruk

**Affiliations:** ^1^ School of Materials Science and Engineering Georgia Institute of Technology Atlanta Georgia USA; ^2^ Air Force Research Laboratory Materials and Manufacturing Directorate Wright‐Patterson AFB Dayton Ohio USA

**Keywords:** interfacial integration, liquid metal electrodes, low‐interfacial noise in wearable electronics, resilient electrophysiological monitoring, thickness‐gradient interfaces

## Abstract

High‐fidelity electrophysiological monitoring requires wearable bioelectronic devices with mechanical properties closely matched to human skin. However, current approaches focus primarily on electrode design and performance, while overlooking mechanical compliance and interfacial continuity with complex and dynamic human skin. Mechanical mismatches across electrodes, connectors, and interconnects often lead to interfacial noise, motion artifacts, and electrical instability. In order to overcome these critical issues, we introduce a thickness‐gradient liquid metal (LM)/SEBS connector that enables seamless interfacial integration with skin, electrode, and interconnector interfaces through gradual mechanical compliance matching and manually activated metal network formation. The activated LM forms continuous and robust conductive networks, maintaining electrical stability under large tensile strain (∼1100%) and for repeatable deformation cycles. Skin‐mimetic conformal integration suggested here suppresses interfacial void formation and stress concentration and thereby preserves stable electrical pathways under dynamic skin deformation. As a result, the proposed system delivers markedly improved signal amplitude and signal‐to‐noise ratio, along with substantially lower noise, relative to conventional connector platforms. By systematically aligning mechanical compliance across sensor components, this mechanically adaptive design preserves seamless electrical pathways and minimizes motion‐induced artifacts under dynamic conditions, providing a robust platform for reliable electrophysiological monitoring in long‐lasting wearable bioelectronics.

## Graphical abstract

Thickness‐gradient liquid metal interfaces with elastomeric substrates enable seamless integration between ultrathin skin‐conformal electrodes and external interconnects. The mechanically compliant connector reduces interfacial mismatch and supports stable ECG/EMG monitoring under bending and motion.

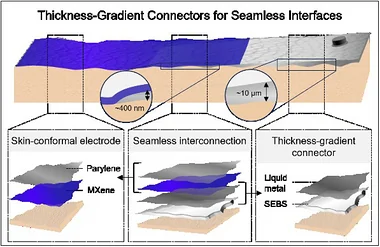

## 1 Introduction

Wearable electronics for electrophysiological signal monitoring offer promising capabilities for continuous, real‐time healthcare and performance monitoring [1, 2, 3, 4]. Accurate acquisition of electrophysiological signals, such as electrocardiography (ECG) [5], electromyography [6], and electroencephalography [7, 8] depends significantly on seamless interfacial integration across multiple interfaces: skin‐electrode, electrode‐connector, and skin‐connector. Any mechanical mismatch at these interfaces can introduce interfacial noise, exacerbate motion artifacts, and decrease the reliability and durability of wearable monitoring devices. Such issues directly impact clinical diagnostics, potentially leading to inaccuracies in diagnosing critical health conditions.

Much research in wearable electrophysiological systems has focused primarily on enhancing the human skin‐electrode interface. Previous studies have developed various high‐sensitivity materials and innovative electrode designs. In particular, dry‐contact electrodes such as thin metallic films [9, 10, 11, 12], conductive polymers [13, 14], carbon‐based materials [15, 16], and composite materials [17], are designed for direct and conformal skin contact. However, these approaches frequently encounter issues such as air gaps, intermittent contact loss, and tribological charging, especially under dynamic conditions and long‐term use, ultimately compromising signal integrity. To overcome these limitations, recent advancements have explored ultrathin electrodes [18, 19], e‐tattoos [15, 20], and smart adhesives [21, 22] aimed at improving skin conformability and enhancing overall signal fidelity.

While significant progress has been achieved at the skin‐electrode interface, these improvements primarily address only the initial stage of signal acquisition. In contrast, the electrode‐connector interface remains an overlooked yet equally critical aspect. Mechanical mismatches between ultrathin electrodes and rigid or thick external connectors commonly result in stress concentrations, mechanical delamination, and instability at their junction. Such instability induces elevated interfacial noise, accelerated signal degradation, and reduced reliability, thus restricting devices largely to short‐term applications rather than supporting sustained, long‐term monitoring.

Commonly employed connector materials include copper, carbon, and conductive tapes, silver pastes [23, 24, 25], and liquid metals (LMs) [6]. Rigid materials such as copper tape and silver paste exhibit excellent electrical conductivity but possess poor mechanical stretchability, resulting in significant interfacial instability during body movements. Carbon tapes offer improved flexibility but at the expense of higher electrical resistance, reducing overall signal quality. Metal evaporation and thin‐film deposition methods partially address adhesion issues but still inadequately resolve conformal skin adhesion. Notably, recent studies have highlighted LMs as a promising connector material due to their high electrical conductivity and intrinsic deformability [26, 27]. This enables mechanically adaptive, skin‐conformal interfaces that remain electrically stable under dynamic body motion.

In this study, we address these critical challenges by introducing a thickness‐gradient LM/styrene‐ethylene‐butylene‐styrene (SEBS) connector materials platform designed to minimize mechanical mismatch across the skin‐electrode, electrode‐connector, and skin‐connector interfaces. The integrated wearable sensor system combines ultrathin skin‐conformal electrodes with mechanically compliant soft connector components [28]. In addition, a button‐type wiring system mechanically decouples the soft, skin‐conformal components from rigid external interconnects, thereby reducing mechanical disturbance transmitted to the electrode‐connector interface during intense and continuous body motion.

The thickness‐gradient connector provides a gradual transition in thickness and mechanical compliance between the ultrathin electrode and the external connection, reducing localized stress concentration, air pocket formation, and maintaining stable interfacial contact during large deformation. Through this mechanically adaptive design, the integrated device preserves electrical continuity across the device‐skin stack and supports stable electrophysiological signal acquisition under both static and dynamic conditions. Epidermal ECG monitoring demonstrates significantly enhanced sensitivity as measured by signal amplitude and signal‐to‐noise ratio (SNR), reduced baseline fluctuation, and improved resistance to motion‐induced artifacts compared with conventional connector platforms.

## 2 Results

### 2.1 Fabrication and Architecture of a Thickness‐Gradient Connector‐Based Device

We prepared a seamless interfacial integration consisting of electrode and connector components designed to minimize mechanical mismatch from the skin interface to the external connection (Figure 1). We first fabricated thickness‐gradient LM/SEBS connectors to achieve mechanically compliant and conformal electrical interfaces (Figure 1a) [29, 30]. A sacrificial layer was first spin‐coated onto a cleaned glass substrate. A SEBS solution was then drop‐cast onto the glass substrate positioned at a tilted angle and allowed to dry, yielding a SEBS film with a continuous thickness gradient formed by gravitational flow during solvent evaporation. The dried LM coating was then manually activated by gently rubbing the surface with a compliant polydimethylsiloxane (PDMS) elastomer. This process generated local shear that ruptured the native oxide shells of the LM particles, allowing the liquid EGaIn cores to spread and coalesce into a continuous conductive network on the SEBS surface after oxidation (see the Experimental Section and Video S1 for details).

**FIGURE 1:**
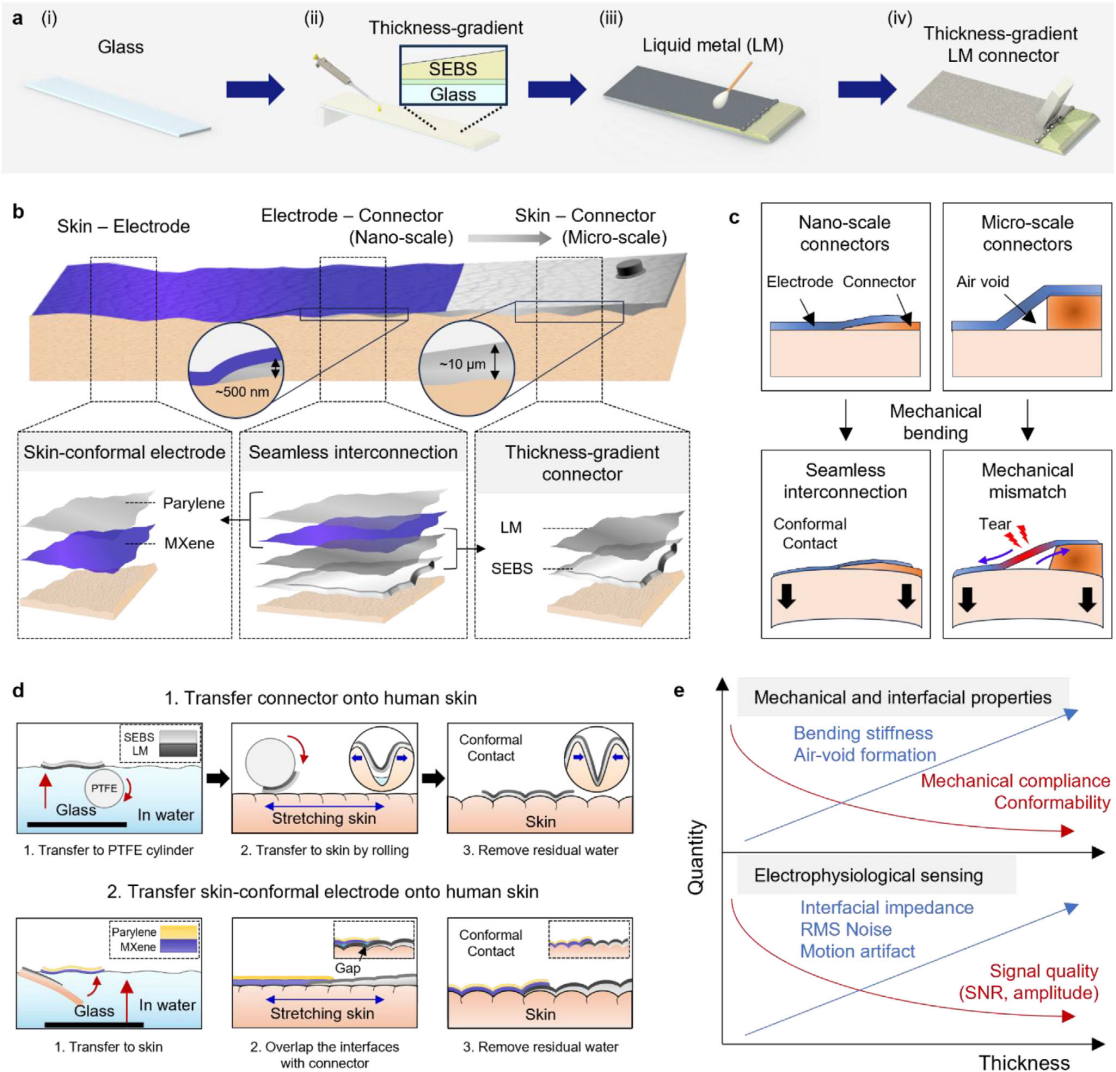
Thickness‐gradient interfacial design for skin‐conformal electrophysiological sensing. (a) Schematic illustrating the fabrication process of thickness‐gradient LM/SEBS connectors. (i) Spin‐coating of a sacrificial layer onto a glass substrate. (ii) Formation of a thickness‐gradient SEBS film by casting the SEBS solution onto an inclined substrate. (iii) Coating the LM slurry onto the dried SEBS film. (iv) Manual shear activation of the LM layer to rupture the oxide shells and form a continuous conductive pathway. (b) Schematic illustration of a thickness‐gradient connector‐based sensory device, which aligns mechanical compliance across device components to enable conformal skin contact. (c) Schematic illustration of the thickness‐gradient connector design enabling void‐free interfacial contact and cooperative deformation by gradually transitioning thickness and mechanical stiffness. (d) Schematic illustrating the transfer process of the LM/SEBS connector (*top*) and skin‐conformal electrode (*bottom*) onto human skin for seamless interfacial integration. (e) Conceptual plots of thickness‐dependent trade‐offs between mechanical and interfacial properties and electrophysiological sensing performance.

To quantify the thickness gradient, we measured the SEBS film using atomic force microscopy (AFM) at multiple positions along the 50 mm gradient direction (Figure S1a–c). The film showed increasing thickness from approximately 500 nm at the thin end to approximately 10 µm at the thick end. The mean thickness profile and sample‐to‐sample variation are presented in Figure S1b. AFM height and phase imaging of the activated LM coating further showed a representative local thickness of approximately 65 nm (Figure S1d,e).

Next, we fabricated nano‐scale skin‐conformal electrodes using a spin‐coating and sacrificial layer‐etching process (Figure S2) (see the detailed fabrication process in the Experimental Section) [31]. We placed the as‐fabricated MXene/parylene electrodes in deionized (DI) water (Type I ultrapure water; 18.2 MΩ cm) to dissolve the sacrificial layer and release them in the free‐standing state. Here, Ti_3_C_2_T*_x_* MXene was used as the conductive electrode material because of its high electrical conductivity, solution processability, and mechanically flexible 2D nanosheet structure in thin films, which make it suitable for soft and conformal bioelectronic sensors [28, 31, 32, 33].

Figure 1b illustrates the overall architecture of a thickness‐gradient connector‐based sensor and the mechanical continuity established across its three primary interfaces. At the skin‐electrode interface, the sensing component consists of an ultrathin crosslinked parylene substrate, a poly‐L‐lysine (PLL) interfacial layer, and a conductive Ti_3_C_2_T*_x_* MXene layer [34, 35, 36, 37, 38]. The approximately 300 nm‐thick parylene support provides sufficient structural integrity for handling while allowing the electrode to conform closely to the microscale topography of the skin. The MXene surface directly contacts the skin for electrophysiological signal acquisition, while the parylene layer mechanically supports the free‐standing electrode.

At the electrode‐connector interface, one end of the MXene electrode overlaps with the thin region of the LM/SEBS connector. This overlapping configuration forms a continuous electrical pathway from the ultrathin sensing region to the soft external connector (∼500 nm‐thick SEBS) without introducing a rigid intermediate component. The LM layer provides deformable conductive contact with the MXene electrode, whereas the SEBS substrate supports the LM network and accommodates induced mechanical deformations. Toward the other end of the connector, the gradually increased approximately 10 µm‐thick SEBS provides greater mechanical support for integration with the traditional button‐type wiring system. Thus, the connector simultaneously provides a highly compliant region near the electrode and a mechanically reinforced region near the external interconnect.

As is known, in conventional micro‐scale connectors, an abrupt transition from the ultrathin electrode to a thick connector creates a pronounced height and stiffness discontinuity, generating air voids [1]. During bending, this discontinuity can prevent complete interfacial contact and concentrate deformation. These localized stresses can promote interfacial lifting, tearing, or delamination. Figure 1c further illustrates how the thickness‐gradient geometry improves mechanical compatibility at the electrode‐connector junction. We suggest that the thin end of the LM/SEBS connector minimizes the geometric discontinuity at the electrode‐connector interface. The connector thickness then increases progressively toward the external wiring region, allowing the electrode and connector to deform cooperatively. This gradual transition maintains conformal, void‐free contact at the edges during bending and distributes deformation over a broader interfacial region. Importantly, the gradient design does not rely on making the entire connector ultrathin. Instead, it combines a thin, compliant interfacial region with a thicker supporting region, thereby balancing interfacial conformability, mechanical durability, and compatibility with external wiring.

It is important to note that this spatially graded architecture distinguishes the present connector from previously reported LM electrodes and stretchable interconnects that primarily exploit the intrinsic deformability and conductivity of liquid metal film, as well as conductive tapes, uniform soft connectors, and hydrogel interfaces that mainly provide electrical routes, bulk stretchability, or skin adhesion [39, 40, 41, 42]. Rather than optimizing a single material property, the present design continuously matches mechanical compliance from the ultrathin electrodes to the mechanically supported external interconnect. This gradual compliance matching reduces abrupt changes in bending stiffness and provides both conformal electrode coupling and mechanically robust external connectivity within a single connector structure.

As shown in Figure 1d, for the water‐assisted transfer assembly, the fabricated LM/SEBS connectors were first immersed in Ultrapure water to dissolve the sacrificial layer and released into a free‐standing state. The free‐standing connectors were then placed onto a cylindrical polytetrafluoroethylene (PTFE) support and transferred onto the stretched skin via a rolling process, enabling uniform contact and conformal adhesion to the epidermis. Separately, the MXene/parylene skin‐conformal electrodes were released from the glass carrier by dissolving the sacrificial layer. Owing to the different hydrophilicity of the parylene (air‐facing) and MXene (water‐facing) surfaces, the released MXene/parylene films floated stably on the water surface without wrinkling [31]. After complete drying, the MXene/parylene electrodes were conformally attached to pre‐stretched skin, resulting in high skin‐electrode contact, interfacial stability, and mechanical durability upon skin release.

For seamless interfacial integration, the free‐standing MXene/parylene electrode was carefully aligned and transferred onto the LM/SEBS connector. One end of the MXene film overlapped with the LM/SEBS connector pad to form a seamless electrical junction between the MXene and the LM. Gentle drying ensured intimate interfacial contact, while the remaining MXene sensing area extended beyond the connector pad to interface directly with the skin. The fully assembled structure was then dried completely, completing the integration of the mechanical gradient interface device.

### 2.2 Effect of Thickness Variation on Interfacial Integration

Figure 1e conceptually illustrates the thickness‐dependent trade‐offs in both mechanical/interfacial properties and electrophysiological sensing performance. From a mechanical and interfacial perspective, increasing thickness leads to elevated bending stiffness and reduced conformability, which promote air‐void formation and interfacial discontinuities. Such abrupt thickness transitions can induce localized stress concentration under deformation, thereby compromising interfacial stability. In contrast, thinner structures exhibit lower bending stiffness and enhanced mechanical compliance, enabling intimate and conformal contact with the skin and adjacent device components.

Although reducing the SEBS thickness improves conformability, a uniformly thin film was not suitable for use as a connector. Indeed, a uniform ∼500 nm SEBS film showed pronounced edge curling and self‐folding during release from the glass substrate (Figure S3). Because of its low bending rigidity, the freestanding elastomeric film was difficult to keep flat and frequently adhered to itself during handling. Similar behavior was observed during transfer onto the skin, making alignment and assembly difficult to control (Figure S4a). The ultrathin film also lacked sufficient mechanical support for integration with the button‐type external wiring.

In contrast, an uniform ∼10 µm film showed the opposite limitation. The thicker film was easier to handle, but its higher bending stiffness reduced its ability to follow the skin surface. In addition, direct contact between the ∼10 µm connector and the skin‐conformal electrode introduced a large thickness mismatch at the electrode‐connector interface, leading to incomplete contact and visible interfacial gaps (Figure S4b). Therefore, the thickness‐gradient connector addresses these two limitations within a single structure. The thin end provides a compliant interface with the ultrathin electrode, while the thicker end improves handling and supports the button‐type connection to external wiring. The gradual thickness transition also avoids the abrupt geometric mismatch associated with a uniformly thick connector.

These tailored mechanical characteristics directly influence electrophysiological sensing performance. Reduced conformability and incomplete interfacial contact can increase susceptibility to mechanically induced signal fluctuations and unstable electrical pathways during deformation. Conversely, mechanically compliant and conformal interfaces facilitate reduced interfacial impedance and suppressed motion artifacts, resulting in improved signal amplitude and higher SNR. Therefore, precise control of device thickness and mechanical compliance is essential for simultaneously achieving robust interfacial integration and stable, high‐fidelity electrophysiological signal acquisition.

### 2.3 Structural Effect of Thickness Gradient Geometry

To investigate seamless interfacial integration on human skin, the fully assembled devices were transferred onto the forearm in Figure 2a. We focused on three mechanically and electrically critical interfaces within the device system: (i) the skin‐electrode, (ii) the electrode‐connector, and (iii) the skin‐connector interfaces. Among these, the skin‐conformal electrode component plays a central role in ensuring signal fidelity, as physiological signals are first acquired at the skin‐electrode interface and subsequently transmitted through the connectors and external wiring to the measurement unit for signal processing, storage, and transmission.

**FIGURE 2:**
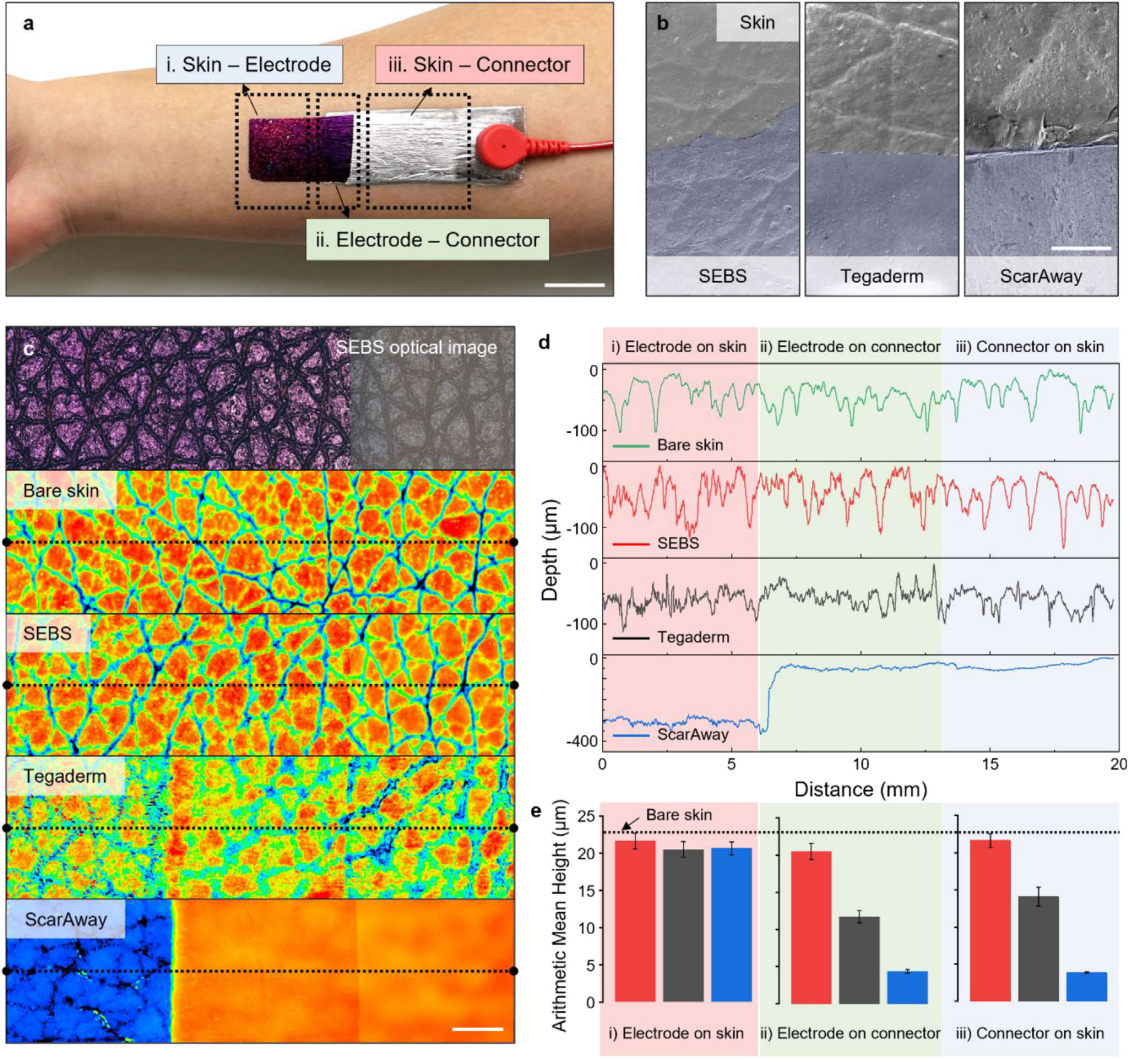
Seamless interfacial integration of skin‐mounted electrophysiological device. (a) Photograph of the assembled sensor transferred onto the human forearm. (i) skin‐electrode, (ii) electrode‐connector, and (iii) skin‐connector (scale bar: 20 mm). (b) SEM images of the device on an artificial skin replica illustrating interfacial conformability with the surface topography and curvature (scale bar: 200 µm). (c) Three‐dimensional surface profiles of the skin replica obtained by laser profilometry for evaluating interfacial conformability (scale bar: 2 mm). (d) Height profile analyses along the dashed lines (20 mm) across the skin‐electrode, electrode‐connector, and skin‐connector interfaces. (e) Quantitative comparison of 3D areal surface roughness (surface area: 1 mm × 1 mm) at each interface.

Furthermore, we compared the LM/SEBS connector with connectors fabricated on common commercially available skin‐contact substrates, including Tegaderm (3 M Company) [43] and ScarAway (Alliance Pharmaceuticals Ltd.) [44], using the same LM coating and device configuration. These substrates were selected as application‐relevant benchmarks because they could be reproducibly assembled into complete devices and represent practical medical‐tape‐based connector platforms. This comparison evaluates the conformability and mechanical performance of fully assembled connector systems rather than isolating the effect of the thickness gradient. When transferred onto an artificial skin replica, the proposed LM/SEBS connectors exhibited highly conformal contact, closely following the surface topography and local curvature of the skin texture (Figure 2b). In contrast, LM on Tegaderm and ScarAway substrates showed limited conformability, failing to adapt to the microscale skin undulations due to their relatively large thicknesses (SEBS: ∼500 nm at the thin end to ∼10 µm at the thick end; Tegaderm: ∼10 µm; ScarAway: ∼200 µm, Figures S5 and S6).

To further quantify interfacial conformability, we investigated the seamless interfacial integration on a skin replica using three‐dimensional (3D) laser microscopy coupled with profilometry height profile analysis (Figure 2c). The LM/SEBS connectors exhibited intimate, continuous contact with the skin replica, with height profiles closely matching the underlying surface topology. By comparison, Tegaderm‐ and ScarAway‐based connectors displayed noticeable gaps and discontinuities at the interface, indicative of incomplete contact and interfacial void formation.

To further examine interfacial conformity across the aforementioned three interfaces, Figure 2d shows height profile analyses (line length: 20 mm, along the *dashed black lines* in Figure 2c). For the LM/SEBS connectors, the surface morphology of the skin replica covered with the device closely resembled that of the bare skin (Figure S7). In contrast, the skin surface covered with Tegaderm exhibited reduced surface roughness, while the ScarAway‐based connectors appeared markedly flat, reflecting non‐conformal coverage due to limited adaptation to the microscale skin topography. A similar trend was observed at the electrode‐connector interface, where the thickness mismatch in Tegaderm‐ and ScarAway‐based connectors led to incomplete contact between the electrode and the connector. This mismatch resulted in the formation of interfacial air voids, which act as mechanically vulnerable regions that concentrate stress under deformation and increase the likelihood of interfacial tearing or failure during bending or stretching.

To quantitatively assess these interfacial differences, three‐dimensional areal surface roughness (Sa) analyses were conducted at each interface in Figure 2e. Using the Sa value of the bare skin replica as a reference (22.3 µm, *dotted black line*), the LM/SEBS connectors exhibited Sa values closely matching those of the native skin across all interfaces. Specifically, at the skin‐electrode interface, the Sa value was 21.6 ± 1.1 µm for LM/SEBS connectors, compared to 20.5 ± 1.1 µm for Tegaderm‐based connectors and 20.6 ± 0.9 µm for ScarAway‐based connectors. At the electrode‐connector interface, the LM/SEBS connectors maintained a high Sa value of 20.5 ± 1.1 µm, whereas Tegaderm‐ and ScarAway‐based connectors showed markedly reduced values of 11.7 ± 0.8 and 4.3 ± 0.2 µm, respectively. A similar trend was observed at the skin‐connector interface, where LM/SEBS connectors exhibited a Sa of 21.6 ± 1.0 µm, while Tegaderm‐ and ScarAway‐based connectors displayed significantly lower surface roughness values of 14.1 ± 1.3 and 3.9 ± 0.1 µm, respectively.

The substantial reduction in Sa for Tegaderm‐ and ScarAway‐based systems indicates surface flattening and incomplete adaptation to the microscale skin topography, reflecting limited conformability and the presence of interfacial gaps. In contrast, the LM/SEBS connectors preserve skin‐mimetic roughness across all interfaces, demonstrating mechanically adaptive and conformal interfacial integration. This suppression of void formation and stress concentration enables stable mechanical coupling and reliable electrical signal transmission during electrophysiological monitoring.

### 2.4 Electrical Characteristics of Seamless Interfacial Integration

For reliable electrophysiological signal acquisition, seamless interfacial integration is essential to maintain stable electrical properties under mechanical deformation of human skin. Disruption at any of these interfaces can lead to increased resistance and degraded signal quality (Figure 3). We first applied LM slurry onto the SEBS substrate, where the LM initially remains as discrete particles distributed across the surface (Figure 3a, *left*, see the detailed procedure in Experimental Section). Upon applying shear‐driven activation, the original particles are mechanically ruptured and spread, forming a continuous and conductive layer anchored to the substrate (Figure 3a, *right*). This activation process transforms the initially discontinuous particle coating into a seamless conductive film.

**FIGURE 3:**
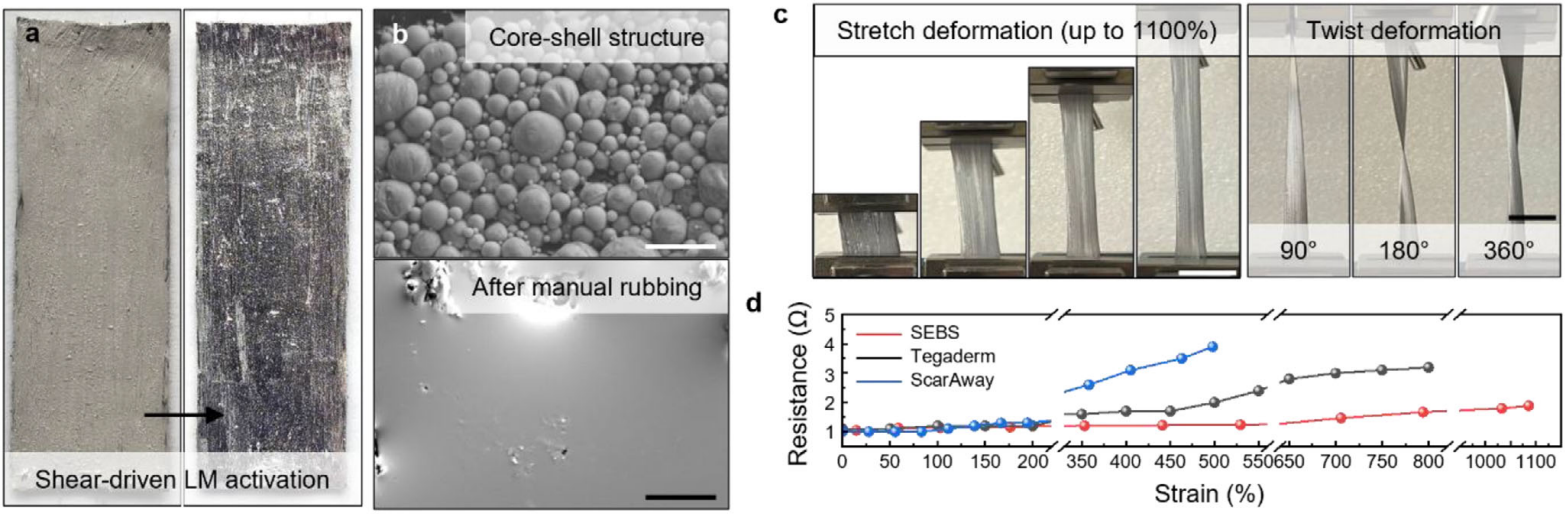
Shear‐driven activation and electrical robustness of LM/SEBS connectors. (a) Optical images of LM on SEBS before (*left*) and after shear‐driven activation (*right*). (b) SEM images of LM particles (*top*, scale bar: 5 µm) and particle rupture after shear‐induced activation (*bottom*, scale bar: 5 µm). (c) Photographs of LM/SEBS connectors under stretching and twisting deformations (scale bar: 10 mm). (d) Resistance variation of shear‐activated LM coated on SEBS, Tegaderm, and ScarAway substrates as a function of applied tensile strain. All LM‐coated substrates maintain relatively stable electrical characteristics with gradual resistance changes up to the onset of substrate mechanical failure, while the LM/SEBS connectors sustain conductivity over a substantially wider strain range (∼1100%) than Tegaderm‐ (∼800%) and ScarAway‐ (∼ 530%) based electrodes.

The underlying mechanism of this transition is attributed to the core‐shell structure of the original LM particles, as confirmed by SEM images (Figure 3b). Prior to activation, the LM particles exhibit well‐defined core‐shell morphologies, where a thin gallium oxide shell encapsulates the liquid EGaIn core, as has been demonstrated in the literature (Figure 3b, *top*) [45, 46]. The particle‐size analysis showed an average particle diameter of 2.0 ± 1.1 µm (Figure S8). In addition, EDX elemental mapping and spectroscopy confirmed the presence of Ga and In as the major constituent elements, while the detected oxygen signal was consistent with the presence of a native surface oxide layer (Figure S9). This oxide shell mechanically stabilizes the particles but electrically isolates neighboring cores. As is known, gentle PDMS rubbing ruptures the native oxide shells of the EGaIn particles, allowing the liquid cores to spread and coalesce into a continuous conductive network (Figure 3b, *bottom*, and Figure S10). EGaIn was selected instead of pure gallium because pure gallium remains solid under typical room‐temperature conditions, limiting conformal coating and shear‐induced particle coalescence [47]. In addition, the gallium oxide shell promotes strong adhesion to the polymer substrate, enabling the LM to remain anchored even after rupture and spreading. This shell rupture, LM release, and oxide‐mediated adhesion collectively enable uniform coverage and mechanically stable conductive pathways, establishing seamless interfacial integration across the substrate surface [26, 48].

The resulting LM‐coated SEBS connectors exhibit excellent mechanical robustness under various deformation modes. Figure 3c illustrates that the LM/SEBS connectors remained intact and continuous during stretching (up to 1100%) and twisting (360°), without visible cracking or delamination. This behavior highlights the ability of the LM to dynamically redistribute strain and maintain conductive continuity.

To quantitatively evaluate electrical stability under tensile deformation, the relative resistance change was measured as a function of applied strain for LM‐coated SEBS, Tegaderm, and ScarAway connectors (Figure 3d). All three connectors exhibited stable electrical characteristics with minimal resistance variation prior to mechanical failure or slip of the underlying substrate. Specifically, the LM/SEBS connectors maintained a resistance change (1.9 Ω at ∼1100% strain), indicating stable conductive pathways even under large deformation. Similarly, LM‐coated Tegaderm and ScarAway connectors also exhibited stable electrical performance with minimal resistance variation over their strain ranges. It is noteworthy that the shear‐activated LM forms continuous and robust conductive networks independent of the mechanical properties and types of substrates, maintaining stable electrical performance up to the point of mechanical failure.

To further evaluate electrical stability under realistic skin deformation conditions, resistance changes were monitored during controlled bending motions (Figure 4a, see the details in Experimental Section). This configuration enables direct assessment of mechanical compliance and electrical continuity in response to curvature‐induced strain.

**FIGURE 4:**
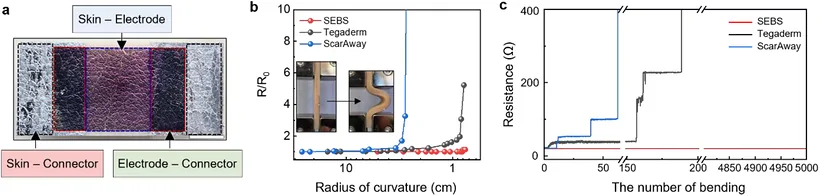
Mechanical compliance and electrical stability of thickness‐gradient LM/SEBS connectors under bending deformation. (a) Optical images of thickness‐gradient LM/SEBS connectors on artificial skin (scale bar: 10 mm). (b) Resistance changes and mechanical integrity of connectors as a function of bending angle (Inset: from flat state on left to bent to a radius of curvature of 5 mm on right). (c) Electrical durability of connectors under 5000 repeated bending cycles.

As is clear from Figure 4b, the Tegaderm‐ and ScarAway‐based connectors exhibited tearing at a radius of curvature of 0.8 and 2.7 cm, respectively. In contrast, the LM/SEBS connectors remained electrically functional up to high bending, such as 0.76 cm of the radius of curvature, exhibiting only approximately 15% resistance change without mechanical failure. Optical images of post‐bending deformation further showed that the LM/SEBS electrode‐connector interface remained comparatively intact, whereas the Tegaderm‐ and ScarAway‐based interfaces exhibited localized lifting, delamination, and mechanical damage (Figure S11). These observations provide evidence that the thickness‐gradient connector is less susceptible to interfacial failure during bending deformation. This superior performance reflects the ability of the compliant SEBS substrate to conform to curved surfaces while maintaining seamless interfacial integration and stable conductive pathways.

To further examine the mechanical integrity of the electrode‐connector junction, a 90° peel test was performed on the electrode attached to the activated LM layer (Figure S12). The MXene‐LM junction exhibited an average apparent peel strength of 11.6 ± 2.2 mN cm^−1^ at a peeling rate of 50 mm min^−1^ (*n* = 3), indicating measurable mechanical retention of the overlapping junction. The measured value is relatively low compared with designed LM composite adhesives, which typically incorporate adhesive polymer matrices or chemical coupling treatments [49]. Nevertheless, it demonstrates measurable mechanical retention of the present ultrathin overlap junction without an additional dedicated adhesive layer.

Next, we assessed the electrical durability of the connectors under repeated bending cycles (radius of curvature: 3.0 cm) on the artificial skin in Figure 4c. Tegaderm‐ and ScarAway‐based connectors exhibited rapid electrical degradation, losing conductivity after 180 and 61 cycles, respectively. In contrast, the LM/SEBS connectors demonstrated exceptional stability over 5000 bending cycles without any resistance variation throughout the cyclic test. This outstanding durability under numerous bending deformations confirms that the thickness‐gradient LM/SEBS connectors enable high mechanical compliance and conformability on the skin, allowing the shear‐activated LM network to maintain stable electrical performance under repeated deformation.

### 2.5 ECG analysis for low‐motion artifacts Under variable conditions

For ECG measurements, a three‐electrode configuration was used, with measurement electrodes positioned on the right (RA) and left arms (LA) and a reference electrode placed on the back of the right hand in Figure 5a [31]. The signals exhibit recurring features that form the PQRST waveform of a single heartbeat, including the P wave, QRS complex, and T wave, which correspond to atrial and ventricular depolarization and repolarization and are used to characterize cardiac electrical activity. ECG measurements were used to evaluate the quality of the skin‐electrode interface and compatibility in the electrode‐connector interface. Figure 5a shows the connector configuration and a representative ECG signal obtained using the proposed system.

**FIGURE 5:**
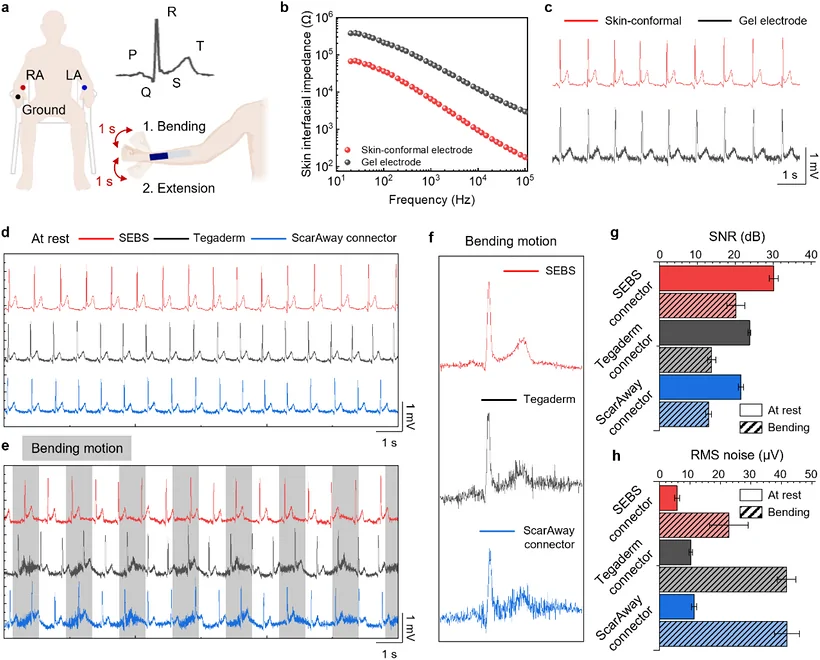
Electrophysiological performance of sensory device incorporating a thickness‐gradient connector. (a) Schematic of the electrode configuration on the human body and a representative ECG signal acquired using the proposed system. (b) Baseline skin‐electrode interfacial impedance measured over a frequency range of 20 Hz to 100 kHz for the proposed electrodes in this study and commercial Ag/AgCl gel electrodes. (c) Representative ECG waveforms comparing signal quality between the proposed electrodes and gel electrodes under static conditions. (d) ECG signals acquired under normal conditions using different connector platforms. (e) ECG signals recorded during periodic bending motion at 1 s intervals. (f) Magnified view of a representative single ECG peak acquired during bending motion. (g) Quantitative comparison of SNR under normal and bending conditions. (h) Root‐mean‐square (RMS) noise analysis under normal and bending conditions.

We first evaluated the skin‐electrode interfacial impedance of the MXene/parylene skin‐conformal electrode to assess its baseline electrical performance relative to a commercial Ag/AgCl gel electrode (Red Dot, 3 M) [31, 50]. As shown in Figure 5b, the MXene/parylene electrode exhibited lower interfacial impedance across the entire measured frequency range (20 Hz to 100 kHz). At 20 Hz, the impedance of the MXene/parylene electrode was 66.8 kΩ, approximately 6 times lower than that of the gel electrode (376.5 kΩ). This comparison demonstrates the robust electrical coupling of the fabricated electrode at the skin interface but is not intended to isolate the effect of the thickness‐gradient connector.

Consistent with this electrode‐level comparison, ECG signals acquired using the MXene/parylene electrode exhibited clear and well‐defined waveform features, including identifiable P, QRS, and T components, with much reduced baseline noise compared with those obtained using the commercial gel electrode under static conditions (Figure 5c). The traditional gel electrode exhibited greater background noise and baseline fluctuation, indicating less stable electrical coupling at the skin interface. These results establish the baseline electrophysiological sensing performance of the MXene/parylene electrode before evaluating the effects of different connector material platforms.

We next evaluated the influence of connector architecture by comparing devices incorporating LM/SEBS‐, Tegaderm‐, and ScarAway‐based connectors while maintaining the same MXene/parylene electrode configuration [43, 44]. ECG signals acquired under normal conditions showed that all connector platforms were capable of capturing physiologically relevant waveform features, including clearly identifiable P, QRS, and T complexes (Figure 5d). This indicates that all connector platforms are capable of acquiring functional electrical contact with the skin under mechanically stable conditions. In particular, the LM/SEBS‐based devices exhibited the highest peak‐to‐peak amplitude of 1.33 mV, representing approximately 13% and 28% higher signal amplitude than Tegaderm‐ (1.18 mV) and ScarAway‐based (1.04 mV) devices. Along with the increased signal amplitude, the LM/SEBS‐based devices showed more stable waveform morphology with reduced baseline fluctuation, indicating more effective electrical coupling and conformal interfacial integration.

The differences in interfacial stability became more pronounced under dynamic mechanical deformation. During periodic bending motion at 1 s intervals (indicated by the gray blocks), the LM/SEBS‐based devices maintained a stable ECG waveform with consistent peak amplitude and minimal baseline fluctuation, whereas Tegaderm‐ and ScarAway‐based devices exhibited pronounced baseline drift and waveform distortion (Figure 5e). A magnified view of a representative single ECG peak acquired during bending further highlights the superior signal integrity of the LM/SEBS‐based devices, which preserved well‐defined waveform features with minimal distortion, whereas Tegaderm‐ and ScarAway‐based devices showed increased noise and reduced peak clarity (Figure 5f). Notably, repeated bending induced mechanical failure at the electrode‐connector interface in conventional Tegaderm‐ and ScarAway‐based devices, resulting in interfacial tearing and partial delamination (Figure S13).

These qualitative observations are quantitatively supported by SNR analysis under both normal and bending conditions (Figure 5g and Note S1). Under normal conditions, the LM/SEBS‐based devices exhibited the highest SNR of 30.1 dB, compared to 23.7 and 21.5 dB for Tegaderm‐ and ScarAway‐based devices, respectively. When subjected to bending deformation, the SNR decreased for all connector platforms due to mechanically induced perturbations. However, LM/SEBS‐based devices maintained a significantly higher SNR of 20.1 dB, whereas Tegaderm‐ and ScarAway‐based devices exhibited substantially lower values of 13.7 and 13.0 dB, respectively. This corresponds to approximately 1.5 times higher SNR, indicating the thickness‐gradient connector design preserves signal quality under dynamic mechanical conditions.

Furthermore, RMS noise analysis revealed superior electrical stability of LM/SEBS‐based devices under both static and dynamic conditions (Figure 5h). Under normal conditions, the LM/SEBS‐based devices exhibited the lowest RMS noise of 5.8 µV, compared to 10.1 and 11.4 µV for Tegaderm‐ and ScarAway‐based devices, respectively. During bending motions, RMS noise increased for all devices; however, the LM/SEBS‐based devices maintained significantly lower noise levels (23 µV) than Tegaderm‐ (42 µV) and ScarAway‐based (43 µV) devices, representing approximately 1.8‐fold lower noise.

These results indicate that the improved ECG performance arises from the combined effects of the thickness‐gradient connector and the manually activated conducting network. The gradual increase in SEBS thickness reduces the abrupt mechanical transition at the electrode‐connector junction and helps maintain conformal contact during deformation, thereby limiting interfacial void formation and localized mechanical disturbance. At the same time, the activated LM layer provides a deformable conductive pathway that preserves electrical continuity under bending. This combination stabilizes the junction resistance and reduces motion‐induced fluctuations, resulting in lower noise and higher SNR under dynamic conditions. Thus, the thickness‐gradient connector provides a mechanically compliant link between the ultrathin skin‐conformal electrode and the external wiring.

Although long‐term wear and sweat tolerance were demonstrated for the skin‐conformal ultrathin electrode alone [31], the integrated LM/SEBS system was not evaluated under these conditions in this study. Sweat may weaken interfacial contact and promote delamination, requiring additional stabilization or moisture‐resistant encapsulation for prolonged use. The sub‐micrometer electrode and connector are intended for single use because repeated removal and reattachment can cause wrinkling or damage. We suggest that further optimization of electrode layout, signal routing, channel isolation, and acquisition hardware will be required for long‐term stability studies.

In this study, though, we conducted a comprehensive comparison of the mechanical and electrical stretchability across all connector systems reported in the literature [15, 51, 52, 53, 54, 55, 56, 57, 58, 59] in comparison to that reported in this study, highlighting the distinct advantage of the thickness‐gradient LM/SEBS design. Here, mechanical stretchability is defined as the maximum tensile strain prior to physical rupture of the connector, whereas electrical stretchability is defined as the maximum tensile strain before electrical failure or loss of conductive continuity [5]. Thus, the relationship between electrical stretchability and mechanical stretchability provides a useful measure of electromechanical coupling: connector systems positioned near the 1:1 trend line (dashed line in Figure 6) can maintain stable electrical conduction until the material approaches its mechanical failure limit, whereas systems located far below this line undergo electrical failure well before mechanical rupture. Therefore, connector systems located toward the upper‐right region of the plot exhibit superior mechanical deformability for elastomeric materials while maintaining usable electrical functionality to the limits.

**FIGURE 6:**
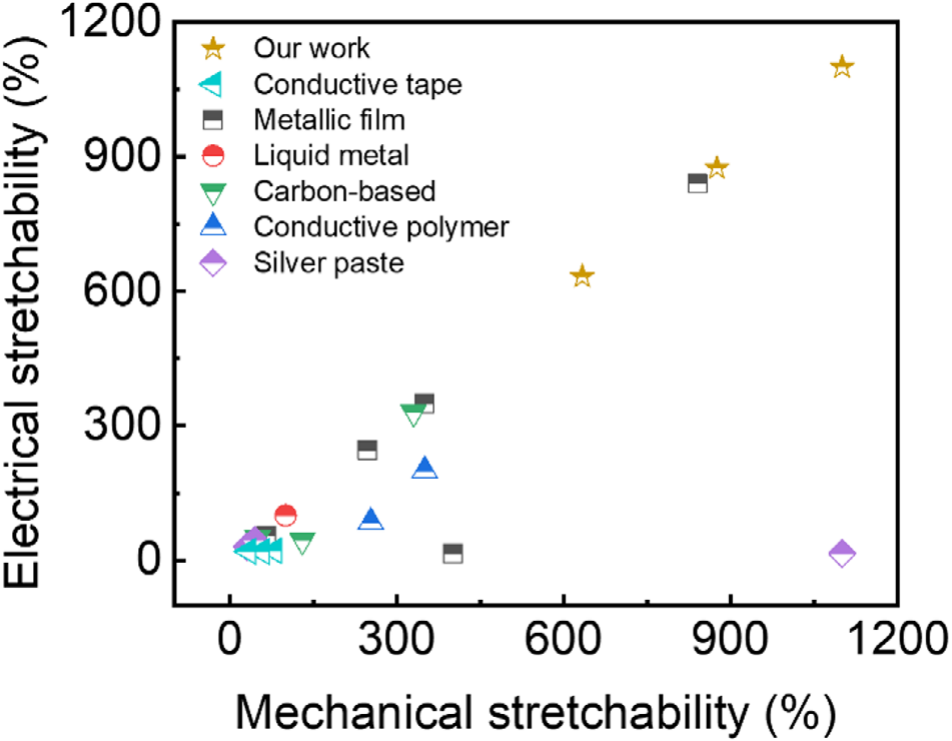
Comparison of mechanical and electrical stretchability of different connector systems, highlighting the superior deformability and conductivity retention of the thickness‐gradient design. The dashed 1:1 line indicates the ideal case in which electrical stretchable ability matches the overall mechanical stretchability of materials to full failure.

The proposed connectors exhibit the highest overall performance, achieving exceptional mechanical stretchability up to ∼1100% while simultaneously maintaining comparable electrical stretchability. The close correspondence between mechanical and electrical stretchability indicates that the conductive LM network remains continuous throughout nearly the entire deformation range of the SEBS connector, with electrical failure occurring only when the connector approaches its mechanical failure limit. This near‐unity electromechanical response distinguishes the thickness‐gradient LM/SEBS connectors from many previously reported systems, where electrical conductivity fails at modest deformations (e.g., see examples of conductive polymers and metal films in Figure 6).

In addition, LM patterned on commercial medical substrates shows moderately high mechanical stretchability (Tegaderm: ∼800%; ScarAway: ∼530%, Figure S14a), confirming that the shear‐activated LM strategy can create stable conductive pathways on different soft substrates. This capability arises from the intrinsically fluidic and highly conductive nature of LM, together with its oxide‐mediated surface adhesion and uniformity, which allow the LM to wet and remain integrated with compliant polymer surfaces. However, their performance remains limited by substrate mechanics, resulting in reduced deformability compared to the LM/SEBS connectors. This result suggests that LM materials can realize their full advantages if properly coupled with a mechanically matched optimized elastomeric substrate.

To decouple the role of substrate and conductor, silver paste coated on SEBS was also evaluated (mechanical stretchability: 1100%, electrical stretchability: 15%). While the SEBS substrate enables large mechanical deformation comparable to LM/SEBS connectors, the electrical stretchability is significantly compromised due to the brittle nature of the silver paste, which leads to rapid resistance increase under strain. This discrepancy indicates that maintaining electrical stretchability requires preservation of conductive percolation under tensile strain, rather than mechanical deformation tolerance alone. This result underscores that substrate compliance alone is insufficient to ensure electrical robustness and that the conductive material must be intrinsically deformable.

Furthermore, commercial conductive tapes exhibit substantially lower mechanical and electrical stretchability, reflecting the limitations of their rigid and non‐compliant material systems, which result in early mechanical failure and loss of conductivity (Figure S14b). A similar trend is observed across broader classes of connector materials reported in the literature. Although conductive polymers, carbon‐based conductors such as CNT‐based electrodes, and thin metallic conductors have progressively improved the balance between deformability and conductivity, many systems still exhibit a noticeable gap between mechanical performance and electrical conductivity stability. This difference frequently indicates that the substrate or structural design may tolerate large strain, but the conductive network often fails earlier.

Overall, the thickness‐gradient LM/SEBS connectors establish a balanced electromechanical design by integrating a highly deformable LM conductor, a stretchable SEBS substrate, and a mechanically compliant gradient architecture. Unlike conventional conductive tapes, brittle silver‐paste conductors, or connector systems, the present design maintains conductive continuity nearly up to the mechanical failure limit of the substrate. This behavior arises from the shear‐activated conductive network, which accommodates large tensile deformation without disrupting electrical percolation. Collectively, these results demonstrate that the record‐high stretchability of the LM/SEBS connectors originates from the synergistic integration of conductive‐network deformability, substrate compliance, and interfacial mechanical stability, rather than the improvement of any single component alone.

## 3 Conclusion

In summary, we have developed thickness‐gradient conductive elastomeric sensors that enable mechanically and electrically seamless interfacial integration for reliable electrophysiological monitoring. By introducing a gradual transition in thickness and mechanical compliance, the proposed system effectively minimizes localized interfacial stress concentration and ensures conformal integration across multiple interfaces, including the skin‐electrode, electrode‐connector, and connector‐skin junctions. The shear‐activated LM network forms continuous and robust conductive pathways anchored to the compliant SEBS substrate, maintaining stable electrical performance under tensile strain, bending deformation, and prolonged cyclic loading.

The thickness‐gradient connector demonstrated exceptional mechanical durability and electrical stability, preserving conductive continuity even under large strain and repeated deformation. Compared with conventional connectors based on medical tapes, the proposed design exhibited superior conformability, reduced interfacial gaps, and enhanced resistance stability, highlighting the critical role of mechanical compliance matching in preserving interfacial integrity. Notably, seamless interfacial integration enabled stable electrophysiological signal acquisition, with significantly improved signal amplitude, higher signal, and reduced motion artifacts under dynamic conditions. These results confirm that mechanically adaptive and conformal interfacial design is essential for maintaining reliable electrical coupling and high‐fidelity electrophysiological signal monitoring.

More broadly, this work establishes a mechanical gradient‐based interface engineering strategy as an effective approach to achieving stable electrical performance and robust interfacial integration in soft bioelectronic systems. By enabling seamless mechanical and electrical continuity across device components, the proposed platform provides a promising foundation for next‐generation wearable and epidermal electronic devices capable of reliable physiological monitoring under dynamic conditions. Suggested designs may also benefit emerging flexible wearable platforms for soft sensors and actuators for wearable sensors at human–machine interfaces by resolving some critical issues associated with robust connections to commercial electrodes [60, 61, 62, 63, 64]. This design framework can be extended to a wide range of bio‐integrated electronic systems requiring mechanically compliant, durable, and reliable interfacial connections.

## 4 Experimental Section

### 4.1 Preparation of LM slurry

Gallium and indium were combined in a ratio of 78.5 wt.% Ga and 21.5 wt.% In to produce EGaIn. Ethanol (240 mL) was added to EGaIn (80 g) and sonicated using an ultrasonication horn with a probe tip diameter of 0.5 inch. Sonication settings included 100% amplitude for 6 min to produce an average particle diameter of 2.0 ± 1.1 µm (n = 240) in Figure S8. The particles were centrifuged, recovered as a pellet, and resuspended in an 85%–87% LM rheology‐modified formulation marketed by ELMNT ST Ink (AeroVironment) [65].

### 4.2 Preparation of MXene (Ti_3_C_2_T_x_) Nanosheets

Ti_3_AlC_2_ MAX precursor was synthesized from high‐purity TiC, Ti, and Al powders (Alfa Aesar) mixed at an atomic ratio of 2:1:1 (TiC:Ti:Al). The powder mixture was uniformly mixed by ball milling for 18 h at 60 rpm using zirconia balls, with a ball‐to‐powder ratio of 2:1. The milled powder was heat‐treated in a tube furnace at 1400°C for 2 h under argon flow (200 cm^3^/min), with 3°C/min heating and cooling rates. The heat‐treated powder was milled with a TiN‐coated bit and sieved to collect particles smaller than 38 µm [66].

Chemical etching was carried out in a 1 L reactor using a mixture of HF, DI water (Type I ultrapure water, 18.2 MΩ cm; Millipore Synergy UV‐R), and HCl (50:150:300 mL). A 50 g of Ti_3_AlC_2_ powder was gradually added into the etchant over 5 min using a screw feeder, and the suspension was stirred at 150 rpm for 24 h while maintaining the temperature at 35°C through water cooling. The etched product was repeatedly washed by centrifugation at 3500 rpm until it reached neutral pH. Delamination was carried out by dispersing the MXene in 1 L of DI water containing 50 g of LiCl, followed by stirring at 150 rpm for 24 h. Subsequent washing cycles were performed until a stable colloidal solution was obtained. To remove multilayered flakes, the suspension was further centrifuged for extended durations, and the resulting MXene dispersion was vacuum filtered through Celgard membranes (64 nm pore size, 3501 coated polypropylene) to produce free‐standing films.

### 4.3 Fabrication of Thickness‐Gradient Connectors

Glass substrates were cleaned by sequential sonication in DI water, acetone, and isopropyl alcohol for 10 min each. The cleaned substrates were then coated with Micro‐90 as a sacrificial layer. SEBS thermoplastic elastomer was dissolved in cyclohexane (ACS reagent, ≥ 99%, Sigma–Aldrich) at a concentration of 5 wt.%. A 2 mL of the SEBS solution was dispensed onto a Micro‐90‐coated glass substrate with dimensions of 2.5 cm × 5.0 cm. To generate the thickness gradient, one end of the substrate was elevated by approximately 1.5 cm using a height‐controlled support structure, with the 6.0 cm dimension aligned along the direction of the slope.

The SEBS solution was dispensed from the elevated end and allowed to flow downward under gravity, producing a continuous thickness gradient with a thinner region at the elevated end and a thicker region at the lower end. The resulting films were dried at room temperature in a desiccator without vacuum. The thickness gradient was controlled and reproduced by maintaining the same SEBS concentration, solution volume, substrate dimensions, tilt height, substrate orientation, and drying conditions for all samples. Under these conditions, the SEBS thickness gradually increased from approximately 0.5 to 10 µm over a distance of 50 mm.

### 4.4 Coating and Shear Activation of LM on the SEBS

The LM slurry was applied across the surface of the dried thickness‐gradient SEBS film and spread into a thin, continuous layer using a PDMS elastomer. The LM slurry was applied over an area of 2.5 cm × 5.0 cm to maintain consistent coating conditions. The coated samples were thermally dried at 60°C for 6 h to remove residual solvent. After drying, the LM‐coated surface was gently rubbed using PDMS elastomer. The applied manual shear ruptured the native oxide shells surrounding the EGaIn particles, allowing the liquid cores to spread and coalesce into a smooth, continuous conductive layer (see the detailed procedure in Video S1) [27].

### 4.5 Transfer Process of Thickness Gradient Connectors and Electrodes Onto Human Skin

Both skin‐conformal electrodes and thickness‐gradient LM/SEBS connectors were released from the glass substrate by dissolving the sacrificial layer in DI water, forming free‐standing electrodes/connectors on the water. Due to the hydrophobicity of the parylene layer and the hydrophilicity of the MXene layer, the electrodes spontaneously oriented themselves with the parylene side facing air and the MXene side in contact with water. Similarly, the hydrophobic SEBS surface faced upward during the water‐assisted transfer. The LM/SEBS connectors floating on the water were first transferred onto a PTFE cylinder (D ≈ 7 cm). The cylinder was slowly immersed in the water and gently rolled to transfer the connectors where the LM side was in contact with the cylinder and the SEBS side facing air. The connectors were then transferred onto the target skin area or commercial artificial replica skin (Deciniee Beauty Co.) by rolling the cylinder across the skin, bringing the SEBS side into direct contact with the skin while leaving the LM side exposed to air.

Next, the skin‐mounted LM/SEBS connector was slowly immersed in water to transfer the skin‐conformal electrodes. During this process, the electrodes floated with the MXene layer facing water and the parylene facing air, allowing the MXene surface to contact the skin upon lifting. The electrodes were partially covered with the LM/SEBS connectors, specifically aligning with the thinner region of the thickness‐gradient. The entire system was fully dried while avoiding mechanical damage like tearing or delamination.

### 4.6 Mechanical and Electrical Measurement

The interfacial impedance of the skin electrodes was measured using an LCR meter (4284A, Agilent Technologies) with a range from 20 Hz to 100 kHz. The impedance of seamless interfacial integration was compared with that of commercial gel electrodes. The skin‐conformal electrodes were fabricated in a 2 cm × 2 cm size. All electrodes were attached to the forearm with a center‐to‐center distance of 5 cm during measurements. For mechanical/electrical stretchability and bending tests, uniaxial tensile tests were performed to evaluate the mechanical properties of the SEBS, medical tapes, and conductive tapes using a universal test machine (EZ‐SX, Shimadzu). The resistance changes were measured by a semiconductor parameter analyzer (4200A‐SCS, Keithley Instruments). The electrical properties were monitored as a function of applied strain to evaluate their retention during stretching and bending.

### 4.7 Evaluation of Electrical Performance Under Bending Deformation

Rectangular connectors (2.0 cm × 3.0 cm) were positioned at both ends of an artificial skin substrate. A rectangular skin‐conformal electrode (4.0 cm × 2.2 cm) was placed between the connectors, with each end of the electrode overlapping the corresponding connector over an area of 1.0 cm × 2.2 cm. The assembled device was mounted on a universal testing machine, and the distance between the two ends of the artificial skin was controlled at a speed of 500 mm min^−1^ to decrease the radius of curvature. During bending deformation, the resistance of the device was continuously monitored in real time using a parameter analyzer. For the cyclic bending test, the devices were repeatedly bent at a fixed radius of curvature of 3.0 cm for 5000 cycles.

### 4.8 Evaluation of ECG Signals of Seamless Interfacial Integration

The thickness‐gradient LM/SEBS connectors and commercial medical tapes were fabricated in rectangular geometry (6.5 cm × 2.5 cm) and were used to evaluate ECG signals robustness against motion artifacts. A commercial wireless electrophysiological data acquisition device (BioRadio, Great Lakes NeuroTechnologies) was used for real‐time ECG signal acquisition. For ECG signal acquisition, two measurement electrodes were mounted on the wrists, with the reference electrode placed on the back of the hand. The sheet resistances of the skin‐conformal electrodes and connectors were approximately 1.5 and 30 Ω sq^−1^, respectively. The detailed ECG signal processing is described in Note S1. The human participant testing was reviewed by the Institutional Review Board of the Institute for Human and Machine Cognition [67] and was determined not to constitute human subjects research. All testing was conducted in accordance with applicable institutional ethical guidelines and the principles of the Declaration of Helsinki, and written informed consent was obtained from the participant prior to device application and ECG measurements.

### 4.9 Characterization

Scanning electron microscopy (S‐3400, Hitachi) imaging was used to characterize the morphology of electrode and connector components. Before imaging, samples were sputter‐coated with Au using a Desk IV sputter coater (Ted Pella Inc.) at 30 mA and 0.05 mbar for 40 s. The thickness of the thickness‐gradient SEBS layer was determined by atomic force microscopy in tapping mode with a scan size of 50 µm × 50 µm, a resolution of 1024 × 1024 pixels, and a scan rate of 0.6 Hz. (Dimension Icon AFM, Bruker) According to usual procedures [68], a profilometry height profile of each interface was characterized by an optical surface profiler (VK‐X3000, Keyence). A semiconductor parameter analyzer was used to measure the resistance of the electrode and connector components.

## Author Contributions

**Jinyoung Kim**: conceptualization, investigation, writing – original draft, methodology, visualization, data curation. **Jaejun Lee**: investigation, validation, methodology, writing – original draft, data curation. **Christopher E. Tabor**: conceptualization, writing – review and editing, methodology, data curation, resources. **Vladimir V. Tsukruk**: conceptualization, writing – original draft, writing – review and editing, project administration, data curation, supervision, resources.

## Conflicts of Interest

The authors declare no conflicts of interest.

## Supporting information




**Supporting File 1**: advs77844‐sup‐0001‐SuppMat.pdf.

**Supporting File 2**: advs77844‐sup‐0002‐VideoS1.mp4.

## Data Availability

The data that support the findings of this study are available from the corresponding author upon reasonable request.

## References

[1] J. Kim , D.‐H. Kang , M. S. Kwak , et al., “Ultrathin Soft Wearable Sensor Materials and Structures: A Review of Current Trends and Prospectives,” ACS Applied Materials & Interfaces 17 (2025): 59994–60027, 10.1021/acsami.5c14329. PMC12598712 41118291

[2] H. C. Ates , P. Q. Nguyen , L. Gonzalez‐Macia , et al., “End‐to‐End Design of Wearable Sensors,” Nature Reviews Materials 7 (2022): 887–907, 10.1038/s41578-022-00460-x. PMC9306444 35910814

[3] K. Mahato , T. Saha , S. Ding , S. S. Sandhu , A.‐Y. Chang , and J. Wang , “Hybrid Multimodal Wearable Sensors for Comprehensive Health Monitoring,” Nature Electronics 7 (2024): 735–750, 10.1038/s41928-024-01247-4.

[4] L. C. Brazaca , A. M. Lozano , B. Mayol , et al., “Advances and Challenges in Wearable Sensors for Health Monitoring,” ACS Applied Materials & Interfaces 18 (2026): 39567–39694, 10.1021/acsami.6c04520. PMC13425566 42460879

[5] H. U. Chung , B. H. Kim , J. Y. Lee , et al., “Binodal, Wireless Epidermal Electronic Systems With In‐Sensor Analytics for Neonatal Intensive Care,” Science 363 (2019): aau0780, 10.1126/science.aau0780. PMC6510306 30819934

[6] Y. Jiang , S. Ji , J. Sun , et al., “A Universal Interface for Plug‐and‐Play Assembly of Stretchable Devices,” Nature 614 (2023): 456–462, 10.1038/s41586-022-05579-z. 36792740

[7] M. Mahmood , D. Mzurikwao , Y.‐S. Kim , et al., “Fully Portable and Wireless Universal Brain–Machine Interfaces Enabled by Flexible Scalp Electronics and Deep Learning Algorithm,” Nature Machine Intelligence 1 (2019): 412–422, 10.1038/s42256-019-0091-7.

[8] L. Tian , B. Zimmerman , A. Akhtar , et al., “Large‐Area MRI‐Compatible Epidermal Electronic Interfaces for Prosthetic Control and Cognitive Monitoring,” Nature Biomedical Engineering 3 (2019): 194–205, 10.1038/s41551-019-0347-x. 30948811

[9] D.‐H. Kim , N. Lu , R. Ma , et al., “Epidermal Electronics,” Science 333 (2011): 838–843, 10.1126/science.1206157. 21836009

[10] H. Yoon , J. Choi , J. Kim , et al., “Adaptive Epidermal Bioelectronics by Highly Breathable and Stretchable Metal Nanowire Bioelectrodes on Electrospun Nanofiber Membrane,” Advanced Functional Materials 34 (2024): 2313504, 10.1002/adfm.202313504.

[11] P. Won , J. J. Park , T. Lee , et al., “Stretchable and Transparent Kirigami Conductor of Nanowire Percolation Network for Electronic Skin Applications,” Nano Letters 19 (2019): 6087–6096, 10.1021/acs.nanolett.9b02014. 31411037

[12] D. Kim , J. Bang , P. Won , et al., “Biocompatible Cost‐Effective Electrophysiological Monitoring With Oxidation‐Free Cu–Au Core–Shell Nanowire,” Advanced Materials Technologies 5 (2020): 2000661, 10.1002/admt.202000661.

[13] R. R. M. Serrano , S. Velasco‐Bosom , A. Dominguez‐Alfaro , et al., “High Density Body Surface Potential Mapping With Conducting Polymer‐Eutectogel Electrode Arrays for ECG Imaging,” Advanced Science 11 (2024): 2301176, 10.1002/advs.202301176. PMC11251564 37203308

[14] S. Yang , Y.‐J. Cao , K. Han , et al., “Stretchable Transparent Electrodes Based on Metal Grid Hybrids for Skin‐Like Multimodal Sensing and Flexible Touch Panel,” Nano Energy 139 (2025): 110942, 10.1016/j.nanoen.2025.110942.

[15] S. Kabiri Ameri , R. Ho , H. Jang , et al., “Graphene Electronic Tattoo Sensors,” ACS Nano 11 (2017): 7634–7641, 10.1021/acsnano.7b02182. 28719739

[16] T. Horii , K. Yamashita , M. Ito , K. Okada , T. Fujie , and N. A. Mater , “Ultrathin Skin‐Conformable Electrodes With High Water Vapor Permeability and Stretchability Characteristics Composed of Single‐Walled Carbon Nanotube Networks Assembled on Elastomeric Films,” NPG Asia Materials 16 (2024): 33.

[17] L. Zhang , K. S. Kumar , H. He , et al., “Fully Organic Compliant Dry Electrodes Self‐Adhesive to Skin for Long‐Term Motion‐Robust Epidermal Biopotential Monitoring,” Nature Communications 11 (2020): 4683, 10.1038/s41467-020-18503-8. PMC7499260 32943621

[18] Z. Yan , D. Xu , Z. Lin , et al., “Highly Stretchable Van Der Waals Thin Films for Adaptable and Breathable Electronic Membranes,” Science 375 (2022): 852–859, 10.1126/science.abl8941. 35201882

[19] R. A. Nawrocki , H. Jin , S. Lee , T. Yokota , M. Sekino , and T. Someya , “Self‐Adhesive and Ultra‐Conformable, Sub‐300 nm Dry Thin‐Film Electrodes for Surface Monitoring of Biopotentials,” Advanced Functional Materials 28 (2018): 1803279, 10.1002/adfm.201803279.

[20] F. Ershad , A. Thukral , J. Yue , et al., “Ultra‐Conformal Drawn‐on‐Skin Electronics for Multifunctional Motion Artifact‐Free Sensing and Point‐of‐Care Treatment,” Nature Communications 11 (2020): 3823, 10.1038/s41467-020-17619-1. PMC7393123 32732934

[21] S. Chun , D. W. Kim , S. Baik , et al., “Conductive and Stretchable Adhesive Electronics With Miniaturized Octopus‐Like Suckers Against Dry/Wet Skin for Biosignal Monitoring,” Advanced Functional Materials 28 (2018): 1805224, 10.1002/adfm.201805224.

[22] H. Min , S. Baik , J. Kim , et al., “Tough Carbon Nanotube‐Implanted Bioinspired Three‐Dimensional Electrical Adhesive for Isotropically Stretchable Water‐Repellent Bioelectronics,” Advanced Functional Materials 32 (2022): 2107285, 10.1002/adfm.202107285.

[23] A. P. Gerratt , H. O. Michaud , and S. P. Lacour , “Elastomeric Electronic Skin for Prosthetic Tactile Sensation,” Advanced Functional Materials 25 (2015): 2287–2295, 10.1002/adfm.201404365.

[24] I. R. Minev , P. Musienko , A. Hirsch , et al., “Electronic Dura Mater for Long‐Term Multimodal Neural Interfaces,” Science 347 (2015): 159–163, 10.1126/science.1260318. 25574019

[25] V. Nair , J. Yi , D. Isheim , et al., “Laser Writing of Nitrogen‐Doped Silicon Carbide for Biological Modulation,” Science advances 6 (2020): aaz2743. 10.1126/sciadv.aaz2743 PMC7442483 32937377

[26] S.‐Y. Tang , C. Tabor , K. Kalantar‐Zadeh , and M. D. Dickey , “Gallium Liquid Metal: The Devil's Elixir,” Annual Review of Materials Research 51 (2021): 381–408, 10.1146/annurev-matsci-080819-125403.

[27] C. J. Thrasher , Z. J. Farrell , N. J. Morris , C. L. Willey , and C. E. Tabor , “Mechanoresponsive Polymerized Liquid Metal Networks,” Advanced Materials 31 (2019): 1903864, 10.1002/adma.201903864. 31403234

[28] J. Kim , G. Jung , S. Jung , et al., “Shape‐Configurable MXene‐Based Thermoacoustic Loudspeakers With Tunable Sound Directivity,” Advanced Materials 35 (2023): 2306637, 10.1002/adma.202306637. 37740254

[29] S. Ozenler , A. A. Alkan , U. S. Gunay , O. Daglar , H. Durmaz , and U. H. Yildiz , “Thickness Gradient in Polymer Coating by Reactive Layer‐by‐Layer Assembly on Solid Substrate,” ACS Omega 8 (2023): 37413–37420, 10.1021/acsomega.3c05445. PMC10568690 37841123

[30] R. L. Davis , S. Jayaraman , P. M. Chaikin , and R. A. Register , “Creating Controlled Thickness Gradients in Polymer Thin Films via Flowcoating,” Langmuir 30 (2014): 5637–5644, 10.1021/la501247x. 24784877

[31] J. Kim , S. Park , J. Jeon , et al., “Robust Skin‐Conformal Nano‐Electrodes for Sustainable Health and Performance Monitoring,” ACS Nano 19 (2025): 30322–30337, 10.1021/acsnano.5c08540. PMC12392733 40796515

[32] D. Song , G. Ye , Y. Zhao , Y. Zhang , X. Hou , and N. Liu , “An All‐in‐One, Bioderived, Air‐Permeable, and Sweat‐Stable MXene Epidermal Electrode for Muscle Theranostics,” ACS Nano 16 (2022): 17168–17178, 10.1021/acsnano.2c07646. 36219847

[33] S. Lee , D. H. Ho , J. Jekal , et al., “Fabric‐Based Lamina Emergent MXene‐Based Electrode for Electrophysiological Monitoring,” Nature Communications 15 (2024): 5974, 10.1038/s41467-024-49939-x. PMC11446925 39358330

[34] N. Driscoll , B. Erickson , B. B. Murphy , et al., “MXene‐Infused Bioelectronic Interfaces for Multiscale Electrophysiology and Stimulation,” Science Translational Medicine 13 (2021): abf8629, 10.1126/scitranslmed.abf8629. PMC8722432 34550728

[35] S. R. Averbeck , D. Xu , B. B. Murphy , et al., “Stability of Ti_3_C_2_T_x_ MXene Films and Devices Under Clinical Sterilization Processes,” ACS Nano 17 (2023): 9442–9454, 10.1021/acsnano.3c01525. PMC11342293 37171407

[36] Y. Gogotsi , MXenes: From Discovery to Applications of Two‐Dimensional Metal Carbides and Nitrides (CRC Press, 2023), 10.1201/9781003306511.

[37] N. Driscoll , A. G. Richardson , K. Maleski , et al., “Two‐Dimensional Ti_3_C_2_ MXene for High‐Resolution Neural Interfaces,” ACS Nano 12 (2018): 10419–10429, 10.1021/acsnano.8b06014. PMC6200593 30207690

[38] M. Naguib , M. Kurtoglu , and V. Presser , “Two‐Dimensional Nanocrystals Produced by Exfoliation of Ti_3_AlC_2_ ,” in MXenes (Jenny Stanford Publishing, 2023), 15–29, 10.1201/9781003306511-4.

[39] Q. Zhuang , K. Yao , C. Zhang , et al., “Permeable, Three‐Dimensional Integrated Electronic Skins With Stretchable Hybrid Liquid Metal Solders,” Nature Electronics 7 (2024): 598–609, 10.1038/s41928-024-01189-x.

[40] L. Tang , S. Yang , K. Zhang , and X. Jiang , “Skin Electronics From Biocompatible In Situ Welding Enabled By Intrinsically Sticky Conductors,” Advanced Science 9 (2022): 2202043, 10.1002/advs.202202043. PMC9376824 35754311

[41] S. Chen , S. Fan , J. Qi , et al., “Ultrahigh Strain‐Insensitive Integrated Hybrid Electronics Using Highly Stretchable Bilayer Liquid Metal Based Conductor,” Advanced Materials 35 (2023): 2208569, 10.1002/adma.202208569. 36353902

[42] D. T. Papanastasiou , S. Harimurti , C. Okuda , et al., “A Novel Thickness‐Gradient Electrospun Nanomesh for Interface‐Free E‐Skin Applications,” Materials Horizons 12 (2025): 4676–4684. 10.1039/d5mh00181a 40215004

[43] 3 M Company , Tegaderm Transparent Film Dressings: Product Profile, 3 M Company, (accessed: May 2026), https://multimedia.3m.com/mws/media/447983O/tegaderm‐transparent‐film‐dressing‐brochure.pdf.

[44] ScarAway, Inc ., ScarAway Silicone Clear Scar Sheets, (accessed: May 2026), https://scaraway.com/products/scaraway‐silicone‐clear‐scar‐sheets/.

[45] N. J. Morris , Z. J. Farrell , and C. E. Tabor , “Chemically Modifying the Mechanical Properties of Core–Shell Liquid Metal Nanoparticles,” Nanoscale 11 (2019): 17308–17318, 10.1039/C9NR06369B. 31513218

[46] W. Kong , N. J. Morris , Z. J. Farrell , R. Wheeler , and C. E. Tabor , “Augmentation of Liquid Metal Particle Mechanics via Non‐Native Oxide Nanoshells,” Advanced Functional Materials 34 (2024): 2308167, 10.1002/adfm.202308167.

[47] M. D. Dickey , “Emerging Applications of Liquid Metals Featuring Surface Oxides,” ACS Applied Materials & Interfaces 6 (2014): 18369–18379, 10.1021/am5043017. PMC4231928 25283244

[48] D.‐H. Kang , J. Kim , S. G. Munoz , J. Jeon , S. Park , and V. V. Tsukruk , “Piezo‐ionic Materials and Structures for Complex Shear Field Monitoring,” ACS Applied Materials & Interfaces 17 (2025): 41350–41360, 10.1021/acsami.5c10061. PMC12278252 40619670

[49] T. A. Pozarycki , D. Hwang , E. J. Barron , B. T. Wilcox , R. Tutika , and M. D. Bartlett , “Tough Bonding of Liquid Metal‐Elastomer Composites for Multifunctional Adhesives,” Small 18 (2022): 2203700, 10.1002/smll.202203700. 36098240

[50] S. Ji , C. Wan , T. Wang , et al., “Water‐Resistant Conformal Hybrid Electrodes for Aquatic Endurable Electrocardiographic Monitoring,” Advanced Materials 32 (2020): 2001496, 10.1002/adma.202001496. 32449249

[51] K. Kim , H. J. Kim , H. Zhang , et al., “All‐Printed Stretchable Corneal Sensor on Soft Contact Lenses for Noninvasive and Painless Ocular Electrodiagnosis,” Nature communications 12 (2021): 1544. 10.1038/s41467-021-21916-8 PMC7943761 33750806

[52] J.‐H. Kim , S.‐R. Kim , H.‐J. Kil , Y.‐C. Kim , and J.‐W. Park , “Highly Conformable, Transparent Electrodes for Epidermal Electronics,” Nano Letters 18 (2018): 4531–4540, 10.1021/acs.nanolett.8b01743. 29923729

[53] S. Choi , S. I. Han , D. Jung , et al., “Highly Conductive, Stretchable and Biocompatible Ag–Au Core–Sheath Nanowire Composite for Wearable and Implantable Bioelectronics,” Nature Nanotechnology 13 (2018): 1048–1056, 10.1038/s41565-018-0226-8. 30104619

[54] D. Li , T.‐R. Cui , J.‐H. Liu , et al., “Motion‐Unrestricted Dynamic Electrocardiogram System Utilizing Imperceptible Electronics,” Nature Communications 16 (2025): 3259, 10.1038/s41467-025-58390-5. PMC11972297 40188239

[55] R. Dong , X. Liu , S. Cheng , et al., “Highly Stretchable Metal–Polymer Conductor Electrode Array for Electrophysiology,” Advanced Healthcare Materials 10 (2021): 2000641, 10.1002/adhm.202000641. 32940002

[56] J. Kim , J. Fan , G. Petrossian , et al., “Self‐Healing, Stretchable and Recyclable Polyurethane‐PEDOT: PSS Conductive Blends,” Materials Horizons 11 (2024): 3548–3560. 10.1039/d4mh00203b 38869226

[57] S. Bagatella , H. Roh , M. Cavallaro , R. Suriano , M. Levi , and A. Gumyusenge , “3D‐Printed Mixed Ionic‐Electronic Conductive Polymer Composites for Long‐Term Bioelectronic Sensing,” Advanced Materials Technologies 10 (2025): 01154, 10.1002/admt.202501154.

[58] S. Boncel , R. G. Jędrysiak , M. Czerw , et al., “Paintable Carbon Nanotube Coating‐Based Textronics for Sustained Holter‐Type Electrocardiography,” ACS Applied Nano Materials 5 (2022): 15762–15774. 10.1021/acsanm.2c03904 PMC9623549 36338322

[59] B. Sun , R. N. McCay , S. Goswami , et al., “Gas‐Permeable, Multifunctional On‐Skin Electronics Based on Laser‐Induced Porous Graphene and Sugar‐Templated Elastomer Sponges,” Advanced Materials 30 (2018): 1804327, 10.1002/adma.201804327. 30306662

[60] Y. Zhao , R. Wu , Y. Hao , et al., “Eco‐Friendly Multifunctional Hydrogel Sensors Enabled Sustainable and Accurate Human‐Machine Interaction System,” Advanced Materials 37 (2025): 2507127, 10.1002/adma.202507127. 40395163

[61] J. He , J. Huang , R. Li , et al., “Hysteresis‐Free and Dynamically Resilient Strain Sensor Enabled by Interfacial Coordination,” Science Advances 12 (2026): aea2450. 10.1126/sciadv.aea2450 PMC12757069 41477844

[62] W. Yang , S. Liu , Z. Wang , et al., “Bioinspired Composite Fiber Aerogel Pressure Sensor for Machine‐Learning‐Assisted Human Activity and Gesture Recognition,” Nano Energy 127 (2024): 109799, 10.1016/j.nanoen.2024.109799.

[63] J. He , P. Xiao , W. Lu , et al., “A Universal High Accuracy Wearable Pulse Monitoring System via High Sensitivity and Large Linearity Graphene Pressure Sensor,” Nano Energy 59 (2019): 422–433, 10.1016/j.nanoen.2019.02.036.

[64] H. C. Xu , Y. Liu , Y. P. Mo , et al., “All‐Fiber Anti‐Jamming Capacitive Pressure Sensors Based on Liquid Metals,” Rare Metals 44 (2025): 4839–4850, 10.1007/s12598-024-03071-3.

[65] AeroVironment , ELMNT, (accessed: August 2026), https://www.avinc.com/solution/elmnt/.

[66] M. Alhabeb , K. Maleski , B. Anasori , et al., “Guidelines for Synthesis and Processing of Two‐Dimensional Titanium Carbide (Ti_3_C_2_T_x_ MXene),” Chemistry of Materials 29 (2017): 7633–7644, 10.1021/acs.chemmater.7b02847.

[67] Florida Institute for Human & Machine Cognition , accessed August 2026, https://www.ihmc.us/.

[68] M. E. McConney , S. Singamaneni , and V. V. Tsukruk , “Probing Soft Matter With the Atomic Force Microscopies: Imaging and Force Spectroscopy,” Polymer Reviews 50 (2010): 235–286, 10.1080/15583724.2010.493255.

