# Supplementary material for: Thickness‐Gradient Liquid Metal Connectors for Seamless Electrophysiological Sensors
Source: Adv Sci (Weinh). 2026 Oct 16:e77844. Online ahead of print. doi: 10.1002/advs.77844 (PMC13674469; doi:10.1002/advs.77844)
Supplement: Supplementary file 1 [file ADVS-9999-e77844-s002.pdf]

WILEY-VCH

Supplementary Information

Thickness-Gradient Liquid Metal Connectors for Seamless Electrophysiological Sensors

*Jinyoung Kim[1]†, Jaejun Lee[1]†, Christopher E. Tabor[2], Vladimir Tsukruk[1]**

[1] School of Materials Science and Engineering, Georgia Institute of Technology, Atlanta, Georgia, 30332, USA

[2] Air Force Research Laboratory Materials and Manufacturing Directorate Wright-Patterson AFB, Dayton, OH, 45433, USA

Supplementary Note 1. ECG Signal Processing modes.

The recorded ECG were sampled at 2000 Hz. Baseline wandering was removed using a 1st-order Butterworth high-pass filter ('butter' function) with cutoff frequency of 0.5 Hz by applying zero-phase filtering in MATLAB ('filtfilt' function). After baseline correction, the signals were separated into two components using zero-phase Butterworth filters: 0.5-100 Hz for the ECG signal band and 100-1000 Hz for the ECG noise band. Under normal conditions, a stable segment of the recording (25-55 s) was selected for analysis. The root-mean-square (RMS) values of the signal band and noise band components were calculated, and the signal-to-noise (SNR) was obtained by the following equation in dB

$$SNR = 20\log\left(\frac{V_{rms,signal}}{V_{rms,noise}}\right)$$

where $V_{rms,signal}$ is the RMS value of the signal, and $V_{rms,noise}$ is the RMS value of the noise.

Under motion conditions, temporal variations in noise level were evaluated using a moving RMS analysis in MATLAB applied to the noise band signal. The RMS was computed using a sliding window of 1500 data points with an overlap of 1000 data points, generating time-resolved RMS trace multiple overlapping segments. The segments representing consistently high average noise levels were selected for analysis.

Supplementary Figures

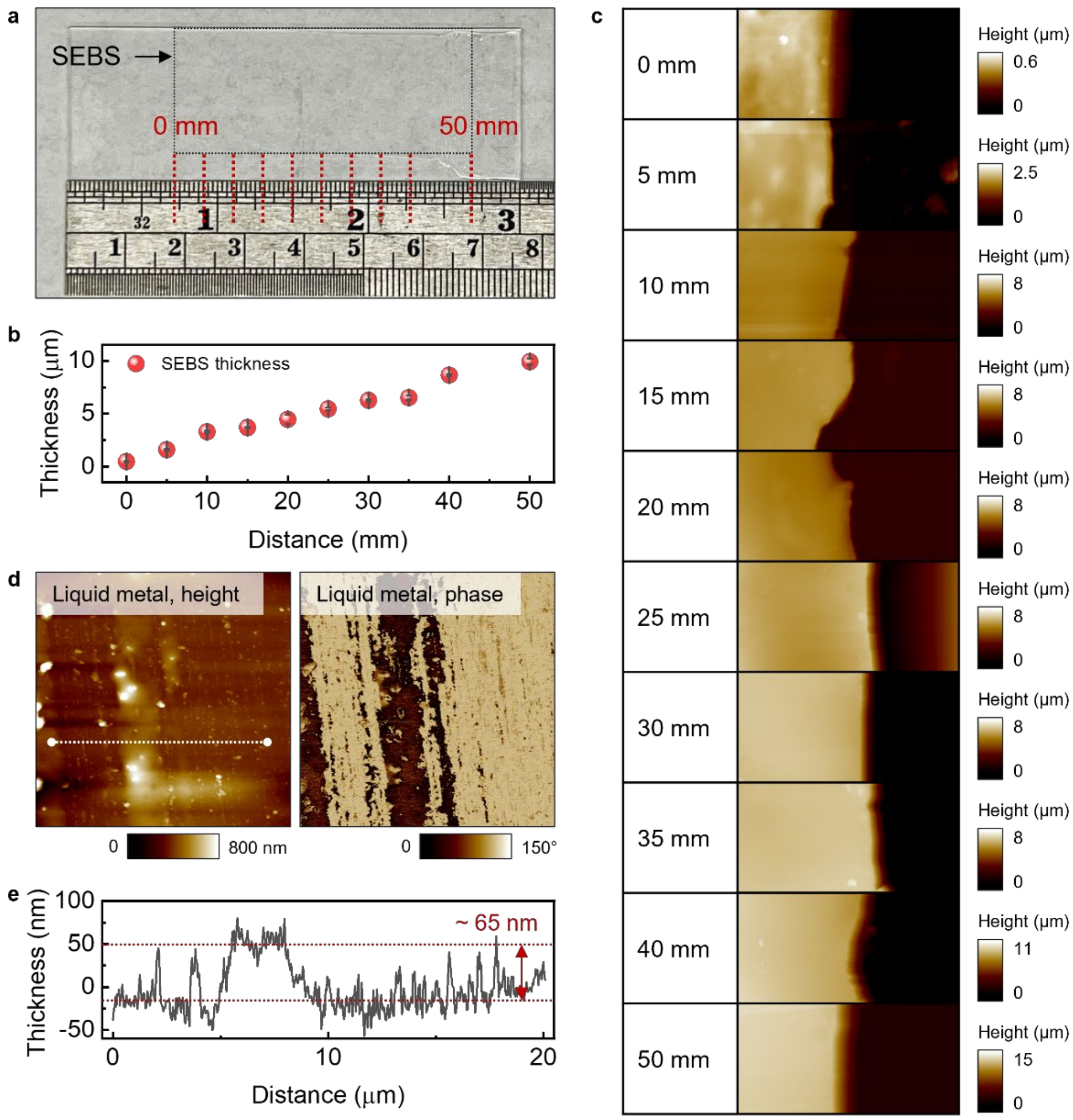

Figure S1. Spatial thickness characterization of the gradient SEBS film and shear-activated LM coating. (a) Photograph of a representative thickness-gradient SEBS film showing the 50 mm gradient region and the positions used for thickness measurements. (b) SEBS thickness as a function of distance along the gradient direction (n = 5). (c) Representative AFM step-height images obtained at positions from 0 to 50 mm along the SEBS film. (d) AFM height and phase images of the shear-activated LM coating. (e) Height profile acquired along the dotted line in panel d, indicating a representative local LM-layer thickness of approximately 65 nm.

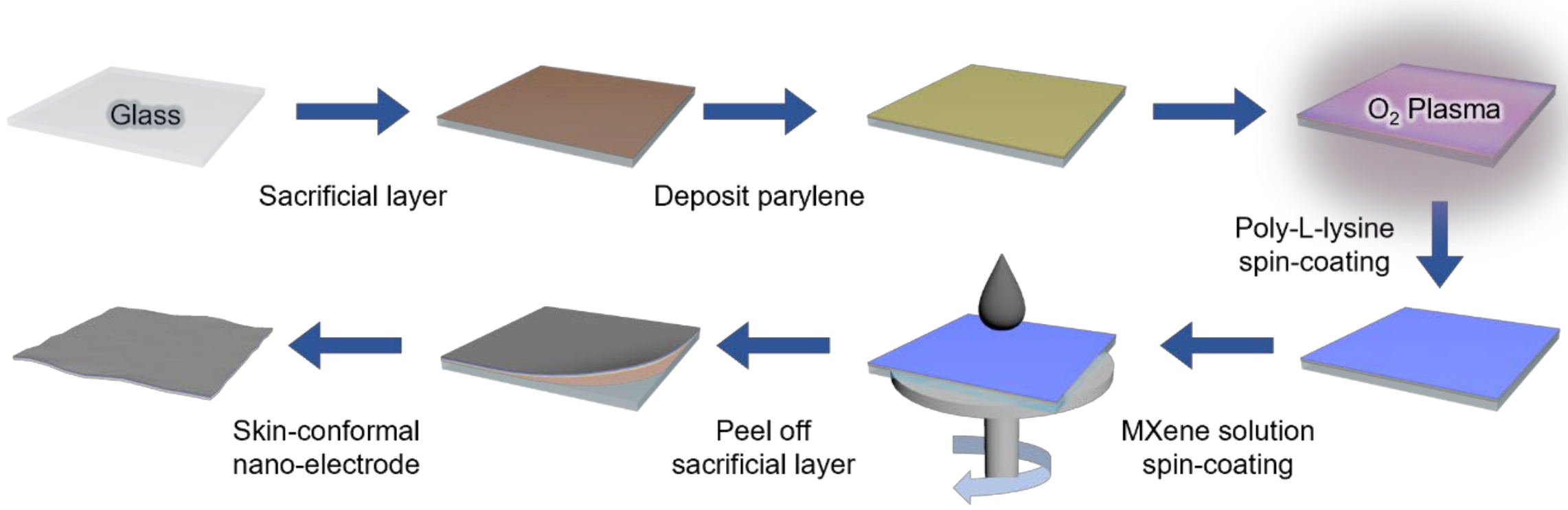

Figure S2. Schematic illustrating the fabrication process of MXene nano-electrodes. (i) Spin-coating a Micro-90 sacrificial layer onto a thoroughly cleaned glass substrate. (ii) Deposition of a parylene film with controlled thickness by chemical vapor deposition. (iii) Oxygen plasma treatment of the parylene surface, followed by (iv) sequential spin-coating of poly-L-lysine (PLL) and (v) MXene layers. (vi) Releasing the MXene/parylene nano-electrode by dissolving the Micro-90 layer in deionized water.[1,2]

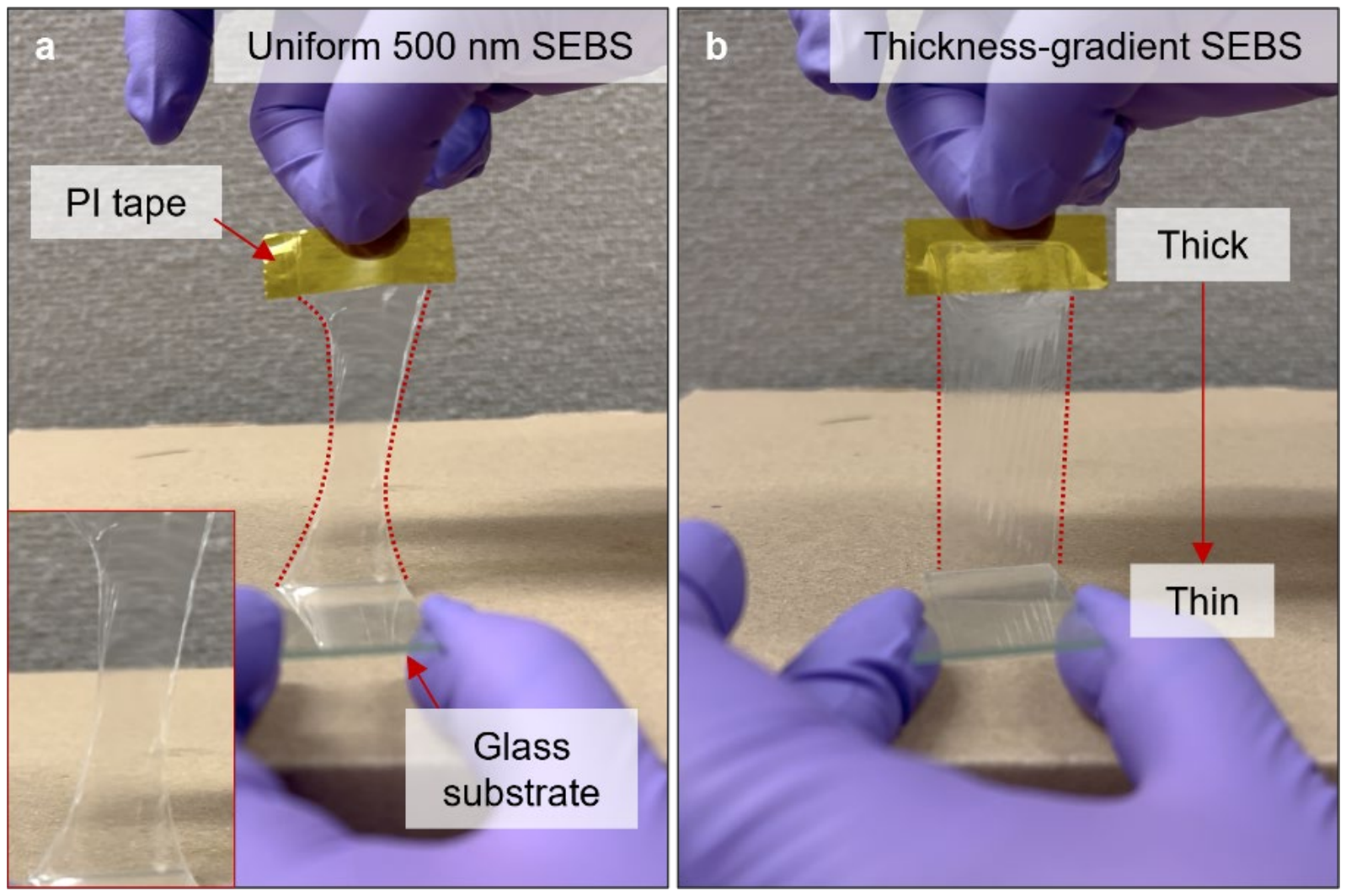

Figure S3. Comparison of the handling and release behavior of uniform ultrathin and thickness-gradient SEBS films. Representative photographs of (a) a uniform ~500 nm SEBS film and (b) a thickness-gradient SEBS film during release from a glass substrate using PI tape.

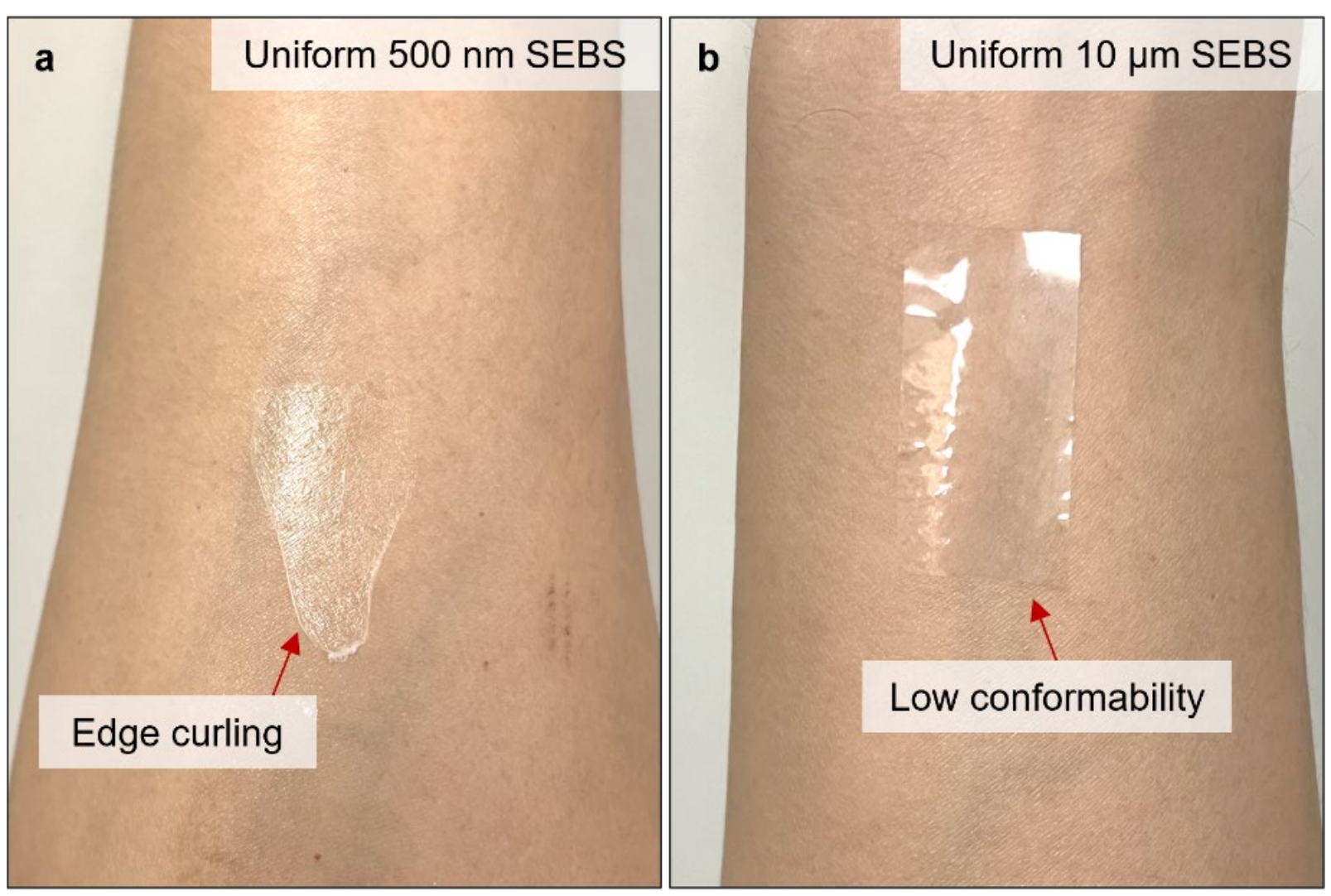

Figure S4. Limitations of uniformly thin and uniformly thick SEBS films during skin integration. Representative photographs of (a) a uniform ~500 nm SEBS film and (b) a uniform ~10 μm SEBS film after placement on the skin.

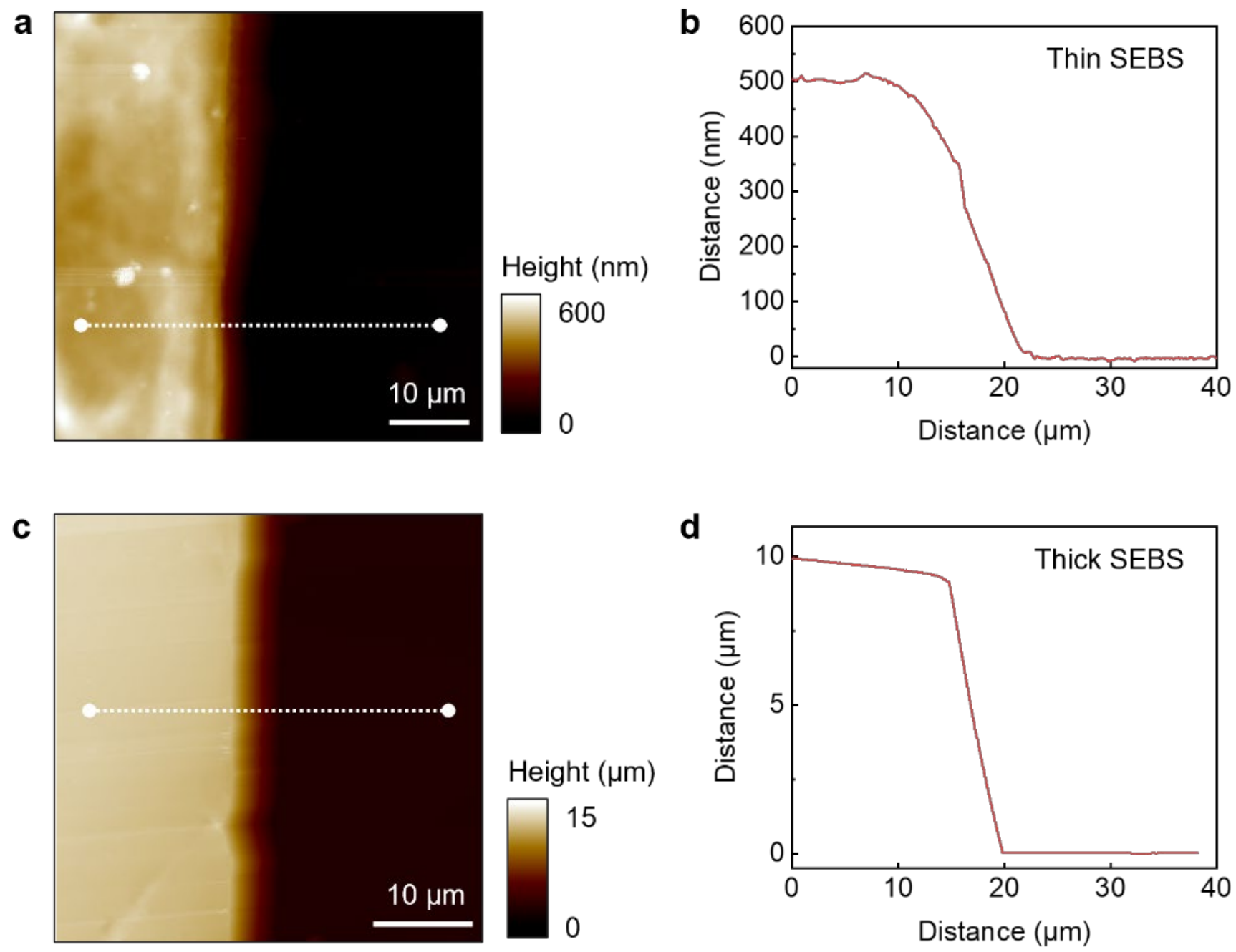

Figure S5. AFM characterization of interfacial conformability of LM/SEBS connectors. (a) AFM topography of a thin LM/SEBS connector. (b) AFM line height profile of the thin LM/SEBS connector. (c) AFM topography of a thick LM/SEBS connector. (d) AFM line height profile of the thick LM/SEBS connector.

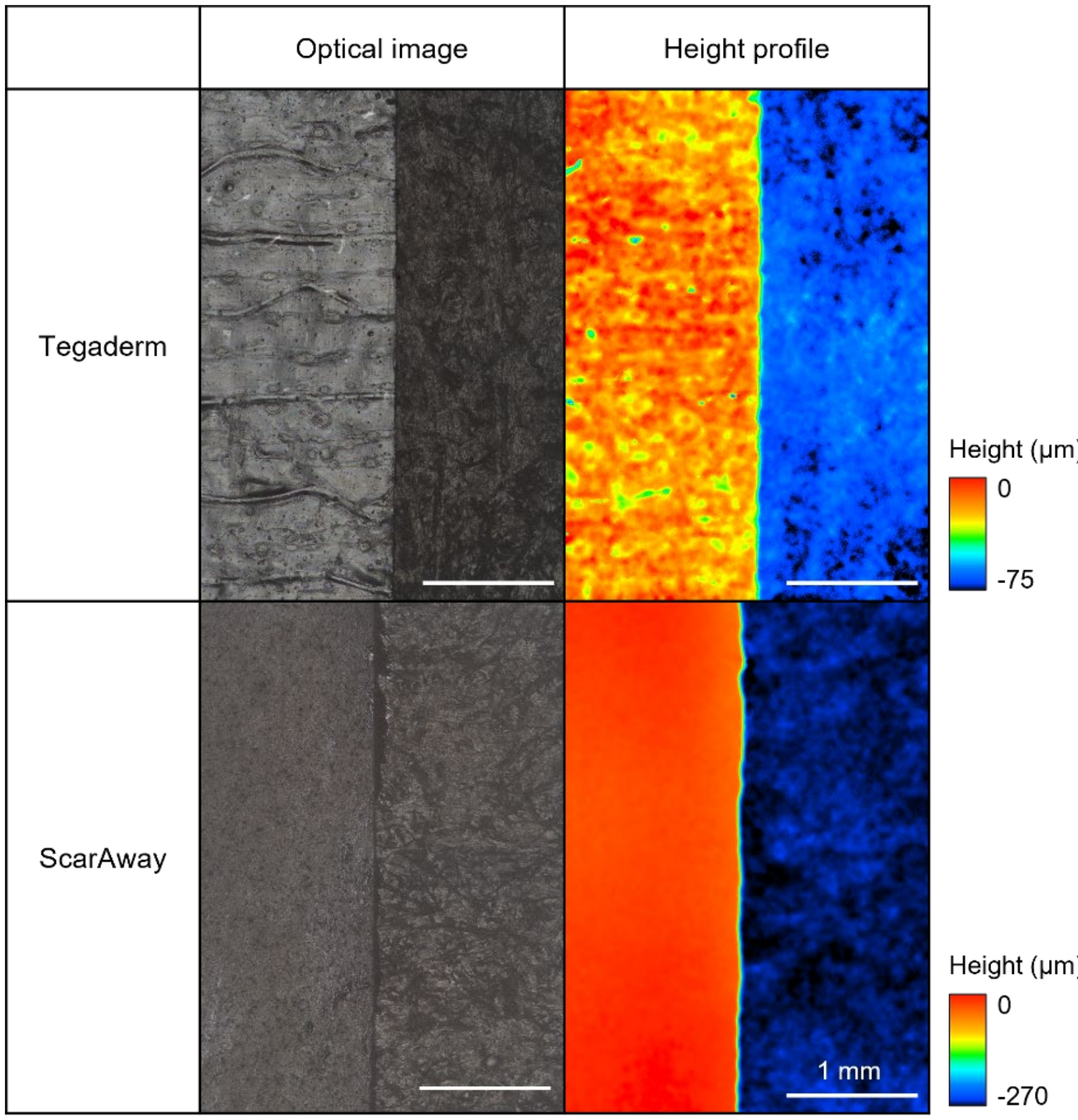

Figure S6. Profilometry analysis of limited conformability for commercial medical tape–based connectors. Optical images and three-dimensional surface height maps obtained by laser profilometry for Tegaderm- and ScarAway-based connectors on an artificial skin replica, illustrating interfacial height mismatch associated with increased connector thickness.

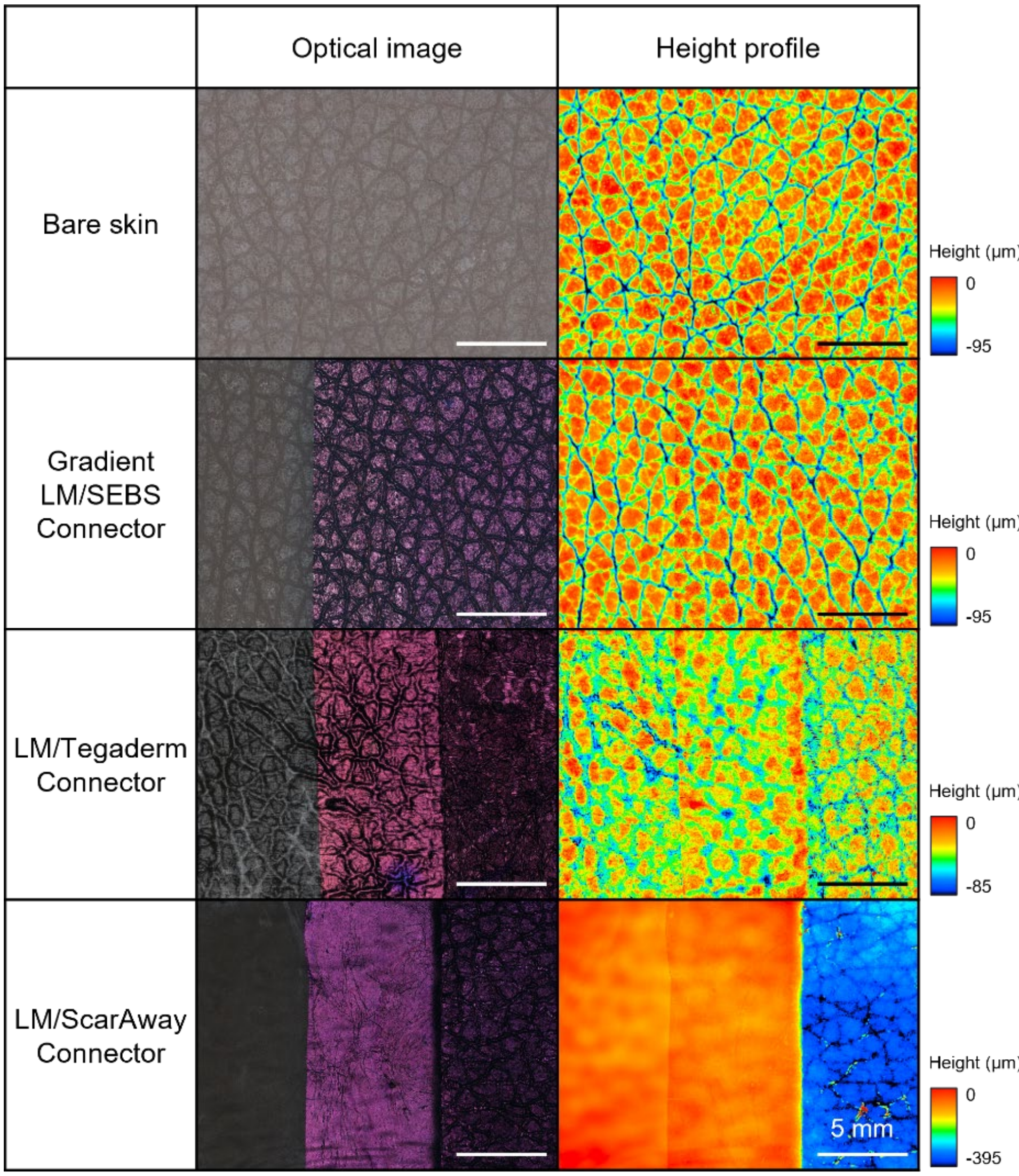

Figure S7. Profilometry mapping for comparison of interfacial surface conformity for different connector systems. Optical images and corresponding three-dimensional surface height maps of a bare skin replica, and gradient SEBS/LM, Tegaderm/LM, and ScarAway/LM connectors.

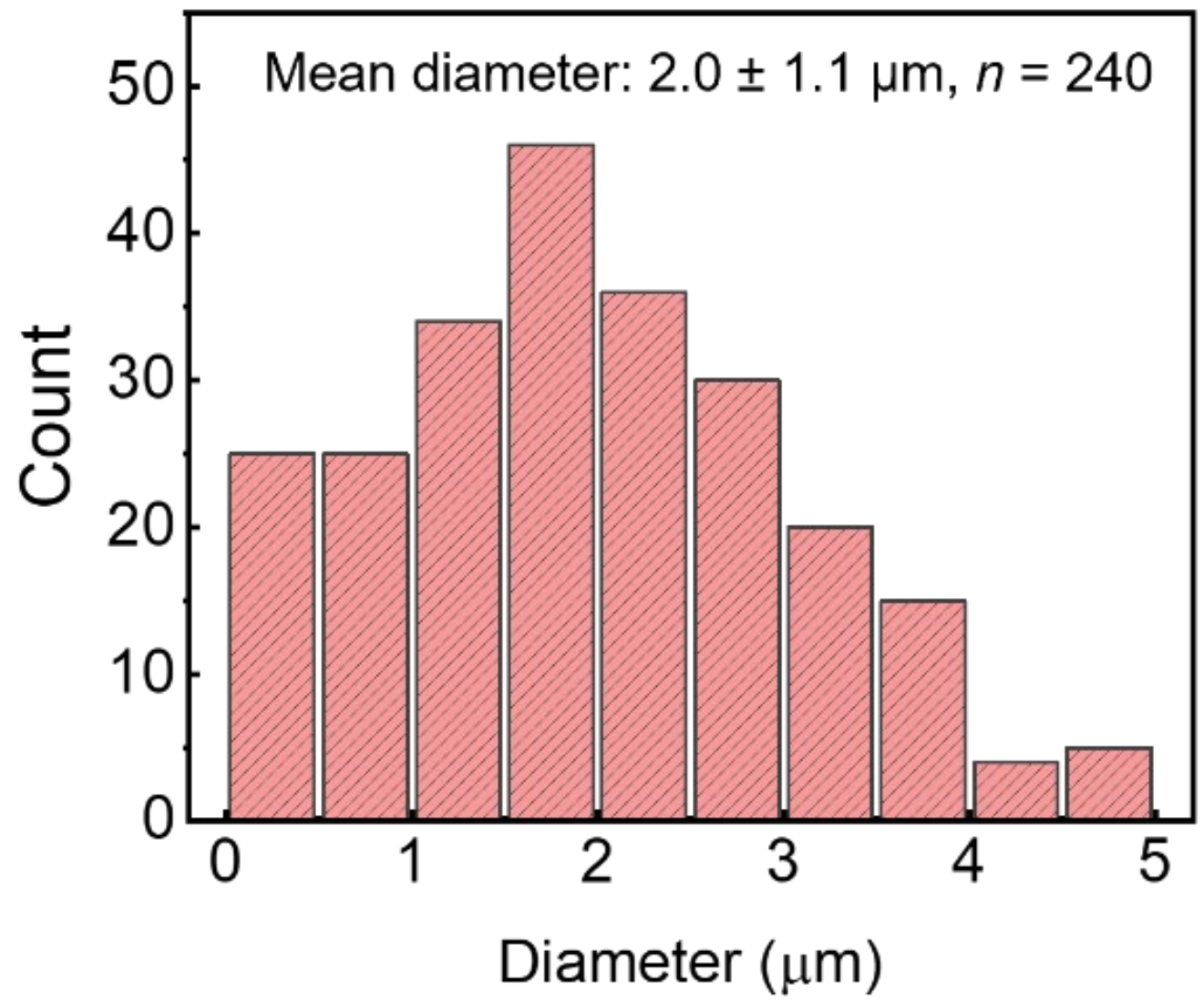

Figure S8. Particle-size distribution of EGaIn particles determined from SEM images. The particles exhibited an average diameter of 2.0 ± 1.1 μm ($n = 240$).

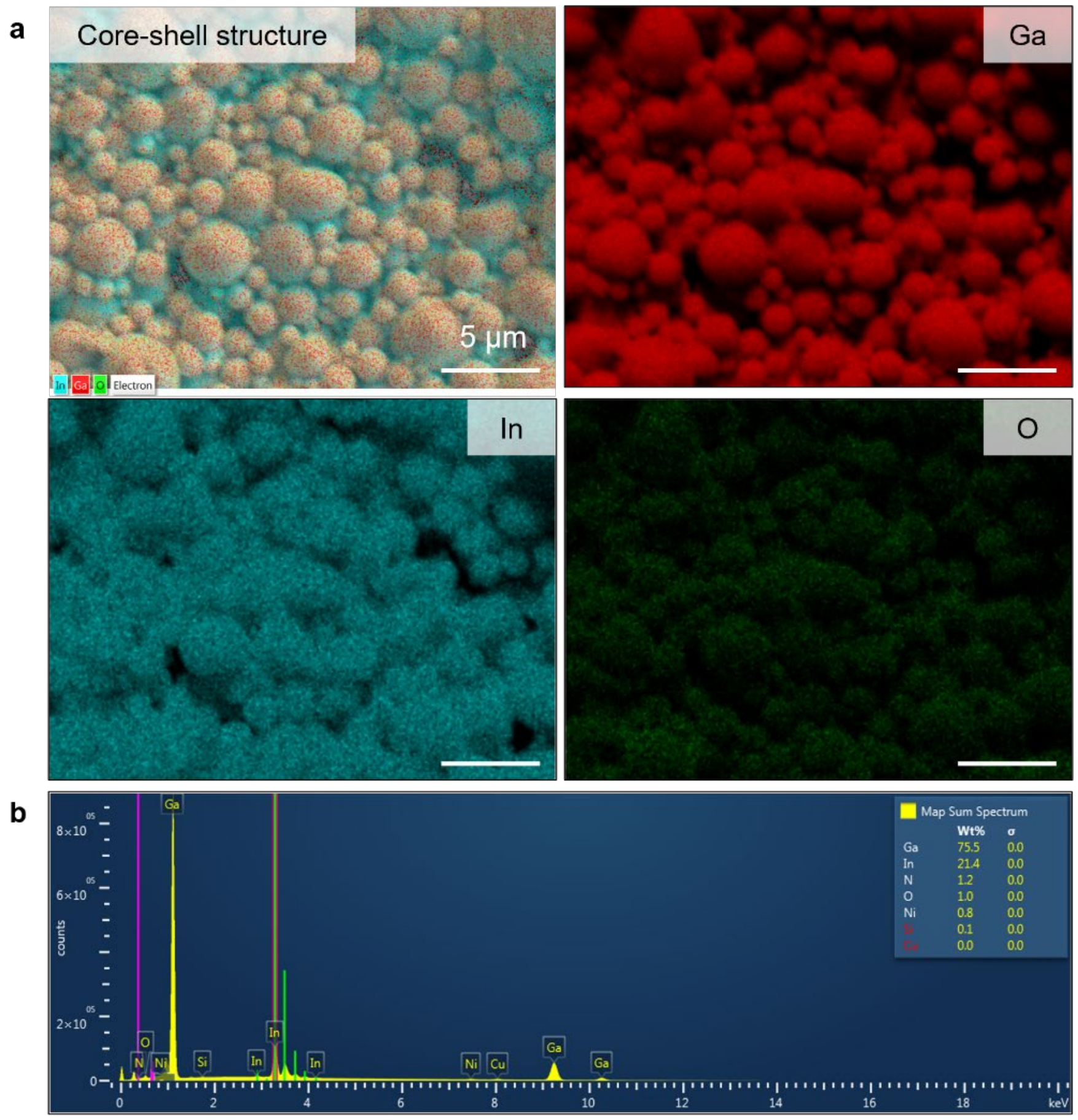

c

Elements	wt%
Ga	75.5
In	21.4
O	1.0

Figure S9. Elemental characterization of as-prepared EGaIn particles. (a) SEM–EDX overlay image and corresponding elemental maps of Ga, In, and O, showing the distribution of the constituent elements within the particles and the presence of surface oxygen associated with the native oxide layer (scale bar: 5 µm). (b) Corresponding EDX spectrum confirming Ga and In as the major elemental components of the LM particles. (c) Table of major elemental components of the particle.

a

b

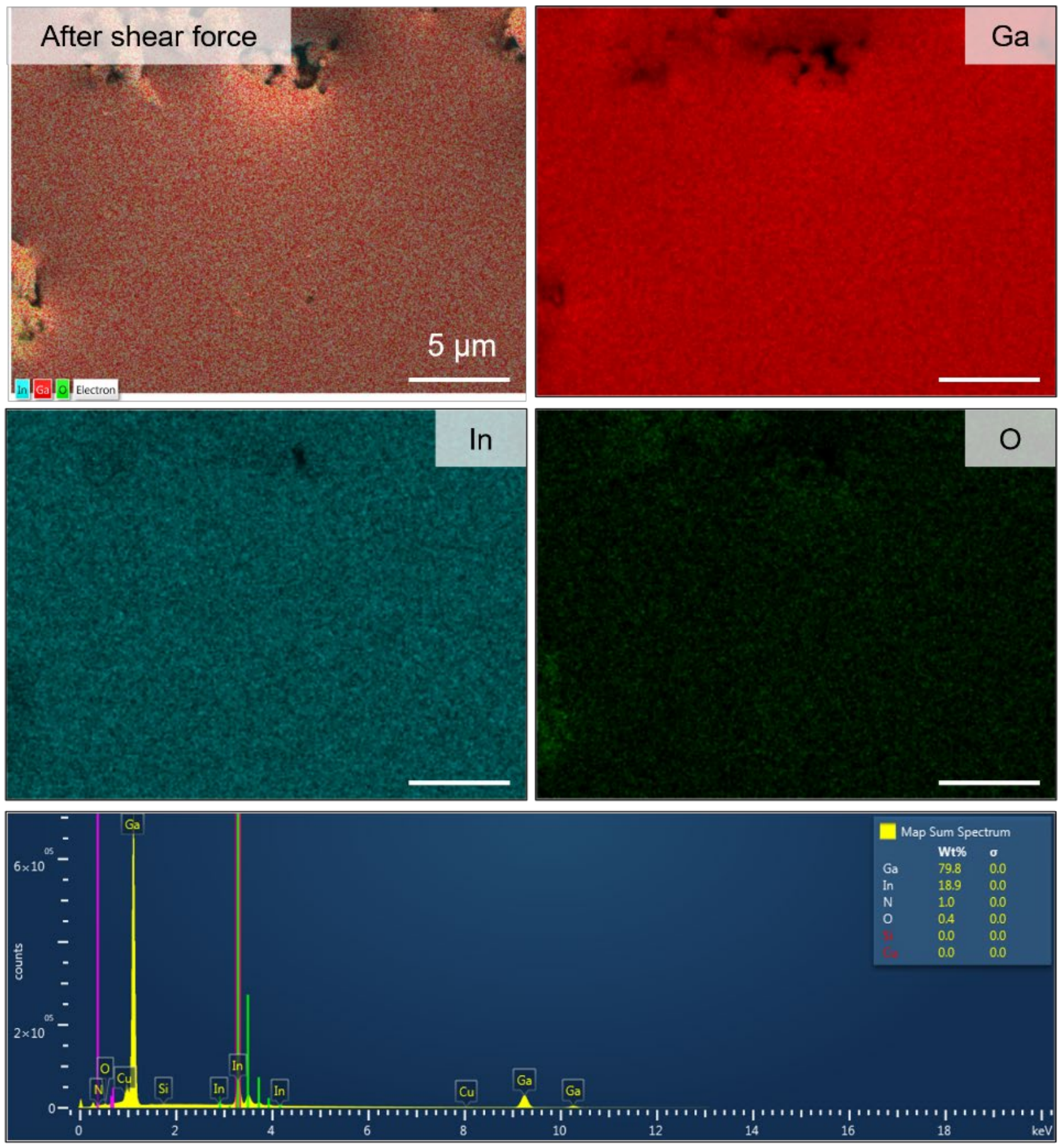

c

Elements	wt%
Ga	79.8
In	18.9
O	0.4

Figure S10. Elemental characterization of particle rupture after shear activation. (a) SEM–EDX overlay image and corresponding elemental maps of Ga, In, and O, showing the distribution of the constituent elements of ruptured LM (scale bar: 5 μm). (b) Corresponding EDX spectrum confirming Ga and In as the major elemental components of ruptured LM. (c) Table of major elemental components of ruptured LM.

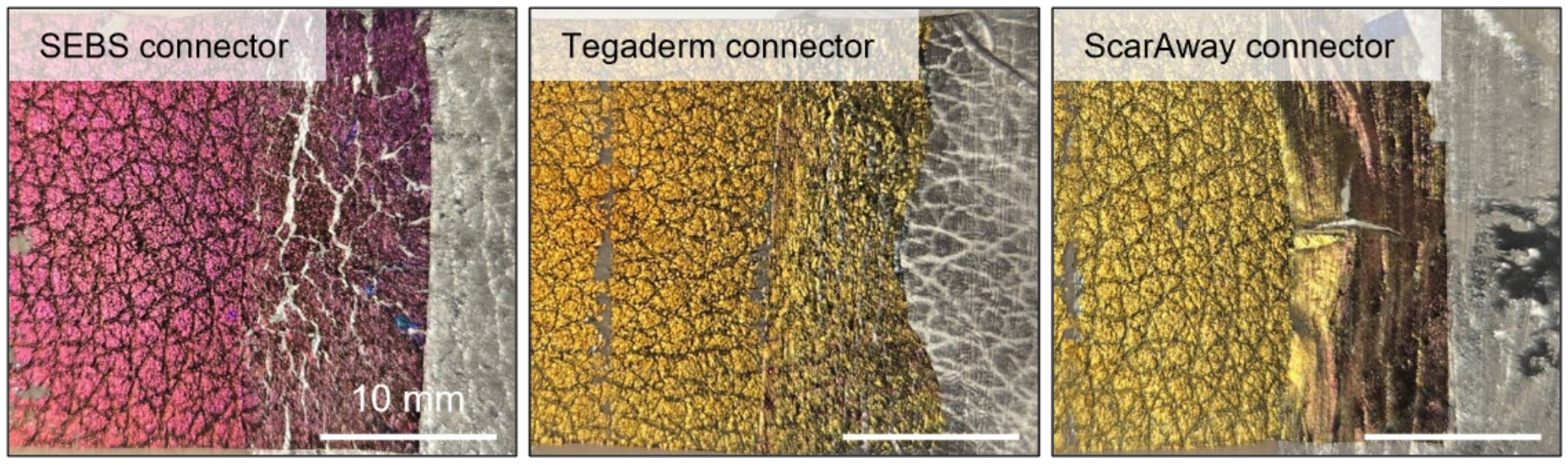

Figure S11. Post-bending integrity of electrode-connector interface. Optical images of the thickness-gradient LM/SEBS, Tegaderm-, and ScarAway-based electrode-connector interface after bending (scale bars: 10 mm).

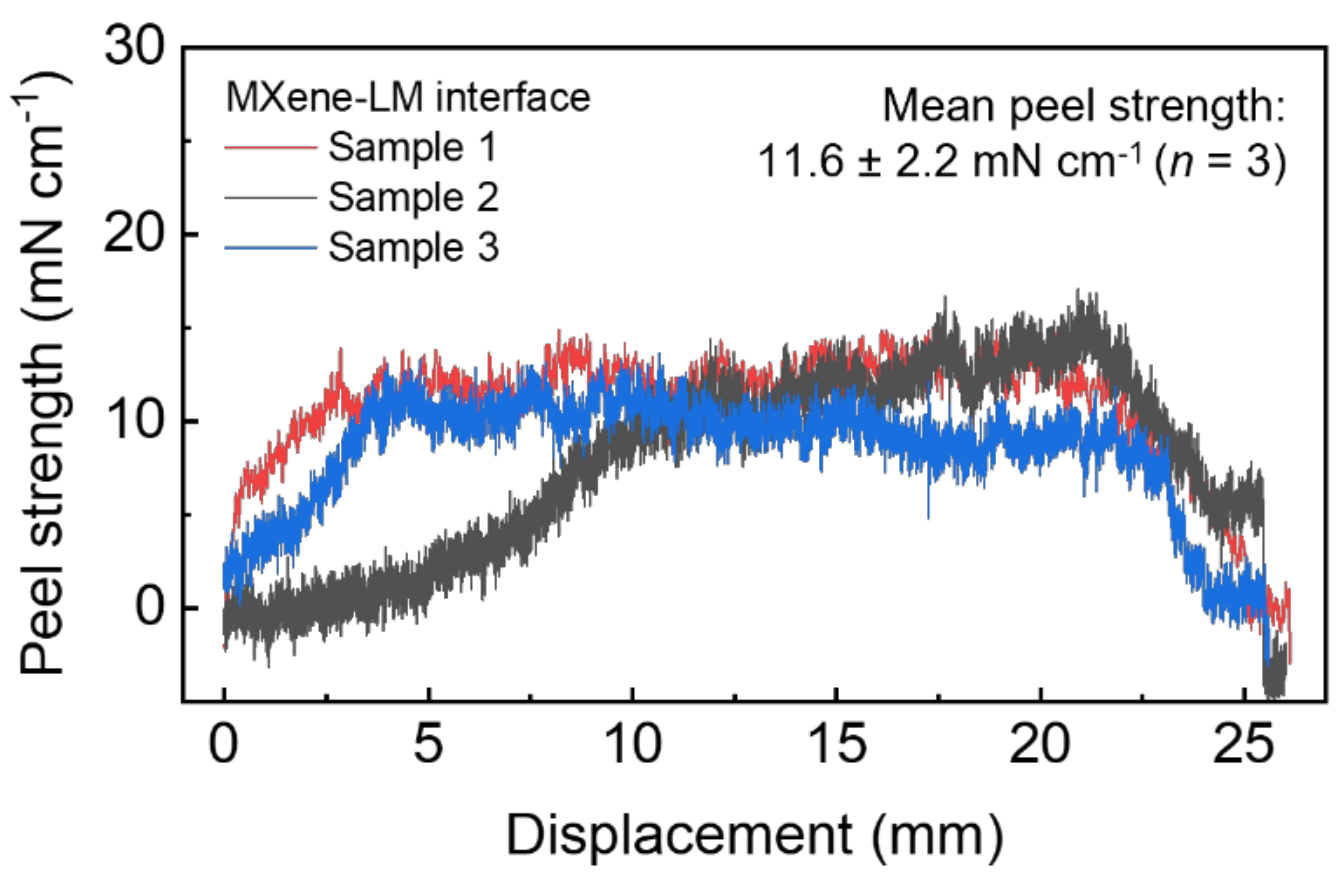

Figure S12. Mechanical characterization of the MXene-LM interface. Peel strength-displacement curves obtained from 90° peel-off tests of MXene/parylene electrodes attached to activated LM on thickness-gradient SEBS. The peel force was normalized by 2 cm peeling width, and the peeling rate was 50 mm min^{-1} (n = 3).

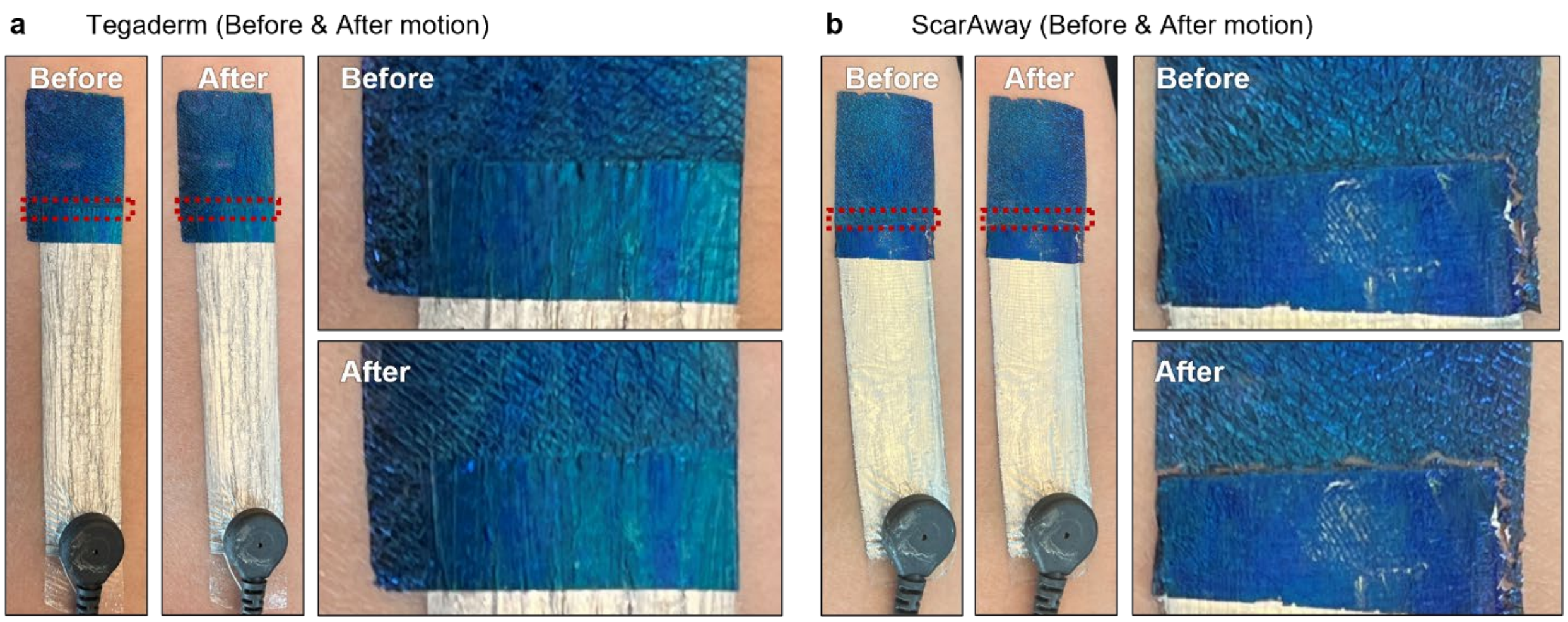

Figure S13. Mechanical degradation of electrode–connector interfaces under repeated bending. Photographs of Tegaderm- and ScarAway-based connectors before and after repeated bending, showing interfacial tearing and partial delamination at the electrode–connector junction.

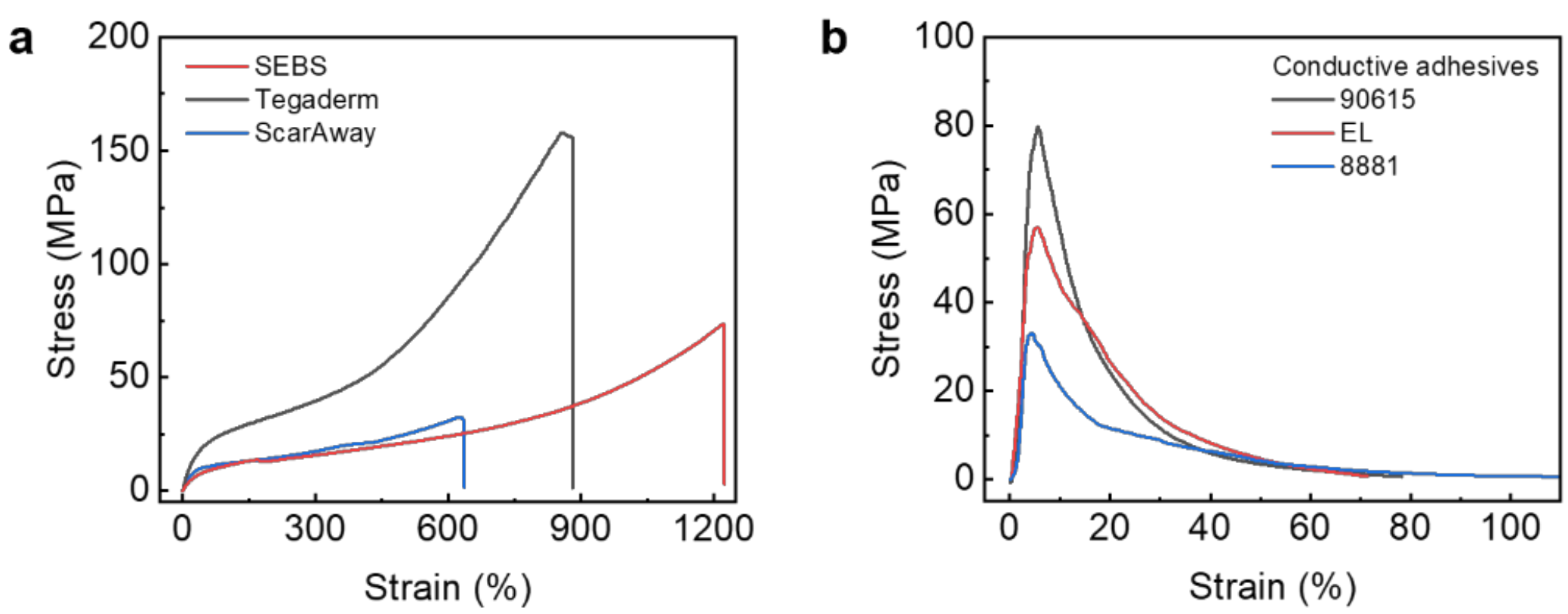

Figure S14. (a) Stress-strain data of LM connectors on commercial medical tapes (Tegaderm and ScarAway) compared with the thickness-gradient LM/SEBS system and commercial conductive tapes (g0615, EL8001774, and 8881).

References

[1] J. Kim, G. Jung, S. Jung, M. H. Bae, J. Yeom, J. Park, Y. Lee, Y. R. Kim, D.-H. Kang, J. H. Oh, *Adv. Mater.* **2023**, *35*, 2306637.

[2] J. Kim, S. Park, J. Jeon, D.-H. Kang, G. M. Bryan, T. J. Broderick, M. Stone, V. V. Tsukruk, ACS Nano 2025, 19, 30322.
